# Supplementary material for: Machine learning prediction of side effects for drugs in clinical trials
Source: Cell Rep Methods. 2022 Dec 7;2(12):100358. doi: 10.1016/j.crmeth.2022.100358 (PMC9795366; doi:10.1016/j.crmeth.2022.100358)
Supplement: Document S1. Methods S1–S3, Figures S1–S5, and supplemental references [file mmc1.pdf]

Cell Reports Methods, Volume *2*

Supplemental information

Machine learning prediction of side effects for drugs in clinical trials

Diego Galeano and Alberto Paccanaro

Supplemental information

Machine Learning Prediction of Side effects for Drugs in Clinical Trials

Diego Galeano, Alberto Paccanaro

Methods S1. Methods implementation and optimization. Related to STAR Methods.

Predictive Pharmacosafety Networks (PPNs)

PPNs[1] represents the drug side effect associations as a bipartite graph and topological features are extracted from the network for the prediction. PPNs model the binary response variable $X_{uj}, u \in \{1, ..., n\}, j \in \{1, ..., m\}$, for n drugs and m side effects, denoting the presence or absence of drug side effect associations. Using Logistic Regression (LR), PPNs modelled this response as a Bernoulli random variable with the following expectation:

$$E[X_{uj}] = \frac{1}{1 + \exp\left(-\sum_s \beta_s Z_s(u, j)\right)} \tag{1}$$

Here, β_s denotes the model parameter and Z_s the model covariate. PPNs extracts network covariates as well as other covariates from intrinsic, biological and taxonomic side information.

Network Covariates

In the following, let u represent a drug node, and j a side effect node. Then, the degree covariates are defined as follow:

$$\begin{aligned} Z_1(u, j) &= \deg(u) \times \deg(j) \\ Z_2(u, j) &= |\deg(u) - \deg(j)| \\ Z_3(u, j) &= \deg(u) + \deg(j) \\ Z_4(u, j) &= \frac{\deg(u)}{\deg(j)} \end{aligned} \tag{2}$$

Here, $\deg(u)$ denotes the degree of node u. The degree product covariate, $Z_1(u, j)$, aims to capture preferential attachment among high-degree drugs and side effects. The degree difference covariate, $Z_2(u, j)$, aims to capture assortativity, i.e. whether high-degree drugs connects to high-degree side effects or to small-degree side effects.

Further, distance covariates accounts for the set of neighbours of drug u, that we denoted as $\mathcal{N}(u)$, and the set of neighbours of side effect j, denoted as $\mathcal{N}(j)$. Let also $J(j, k)$ denotes the Jaccard similarity between the neighbours sets $\mathcal{N}(j)$ and $\mathcal{N}(k)$, which is defined as follow:

$$J(j, k) = \frac{|\mathcal{N}(j) \cap \mathcal{N}(k)|}{|\mathcal{N}(j) \cup \mathcal{N}(k)|} \tag{3}$$

The Jaccard-based covariates quantify structural similarity between drug pairs and side effect pairs:

$$\begin{aligned} Z_5(u, j) &= \max_{k \in \mathcal{N}(u) - \{j\}} \{J(j, k)\} \\ Z_6(u, j) &= \max_{k \in \mathcal{N}(j) - \{u\}} \{J(u, k)\} \end{aligned} \tag{4}$$

Finally, Jaccard-based predictors based on Kullback-Leibler divergence ($\mathcal{K}_{\mathcal{L}}$) between the overall distribution of similarities between a drug ($\bar{D}_{se}$) and the drugs in its local neighbourhood ($D_{se}(u, j)$) or between side effects ($\bar{D}_{se}$) and side effects in its neighbourhood ($D_{se}(u, j)$) are defined as follow:

$$\begin{aligned} Z_7(u, j) &= \mathcal{K}_{\mathcal{L}}(D_{se}(u, j), \bar{D}_{se}) \\ Z_8(u, j) &= \mathcal{K}_{\mathcal{L}}(D_{drug}(u, j), \bar{D}_{drug}) \end{aligned} \tag{5}$$

Intrinsic, biological and taxonomic covariates

Given additional information about drugs (or side effects), these can also be included in the prediction. Intrinsic covariates are those that are defined based on the intrinsic properties of drugs, i.e. chemical structure. For instance, let $d_{chem}(u,k)$ represent a chemical distance between two given drugs u and k, then, the intrinsic covariate is defined as:

$$\begin{aligned} Z_9(u,j) &= \min_{k \in \mathcal{N}(j)-\{i\}} \{d_{chem}(u,k)\} \\ Z_{10}(u,j) &= \min_{k \in \mathcal{N}(j)-\{i\}} \{d_{DT}(u,k)\} \\ Z_{11}(u,j) &= \min_{k \in \mathcal{N}(j)-\{i\}} \{d_{ATC}(u,k)\} \\ Z_{12}(u,j) &= \min_{k \in \mathcal{N}(j)-\{i\}} \{d_{MedDRA}(u,k)\} \end{aligned} \tag{6}$$

We also use the other covariates presented in [1]. These include (i) intrinsic, defined by our 2D Tanimoto chemical similarity; (ii) biological, defined by drug target similarity and (iii) taxonomy, defined by our ATC and MedDRA taxonomy similarities. For PPNs, all the similarities were converted into distances by subtracting one from each similarity matrix.

Optimisation We use the Matlab built-in function `mnrfit` (link function `logit`) to learn the coefficients estimates (β_s) of the multinomial logistic regression of the nominal responses in X on the predictors in Z.

Hyperparameters tunning PPNs does not require hyperparameters tunning.

Low-rank matrix factorization (MF)

The MF model[2] is based on the assumption that the binary matrix $X \in R^{n \times m}$ for n drugs and m side effects of rank $k \ll min\{m,n\}$, can be expressed as the product of two matrices of rank-k, as follow:

$$X \approx PQ \tag{7}$$

here $P \in R^{n \times k}$ and $Q \in R^{k \times m}$. This model amounts to assign a low-dimensional feature vector to each drug and a low-dimensional feature vector to each side effect such that the dot-product between the features vectors is related to the probability that a drug is associated to a side effect. The rank of X is k — the number of features of each drug and side effect. The matrices P and Q are the solution of the following optimisation problem:

$$\min_{P,Q} \mathcal{L}(P,Q) = \underbrace{\frac{1}{2}\|X - PQ\|_F^2}_{\text{low-rank model}} + \underbrace{\frac{\lambda}{2}(\|P\|_F^2 + \|Q\|_F^2)}_{\text{regularisation}} \tag{8}$$

where $\|.\|_F$ is the Frobenius, and the term $\lambda(\|P\|_F^2 + \|Q\|_F^2)$ is a $\mathcal{L}_2$ regularisation term with penalty $\lambda > 0$, which is added to the objective function in order to prevent overfitting.

The problem is non-convex in both P and Q. Gradient descent-based optimisation methods are required to approximate the solution. Thus, the derivatives of (8) are:

$$\frac{\partial \mathcal{L}(P,Q)}{\partial P} = -(X - PQ)Q^T + \lambda P \tag{9}$$

$$\frac{\partial \mathcal{L}(P,Q)}{\partial Q} = -P^T(X - PQ) + \lambda Q \tag{10}$$

Optimisation We used the code provided by the authors[2]. The optimisation was done using conjugate gradient descent (CGD) and approximated line searches based on polynomial interpolation with Wolfe-Powel conditions to find the local minima in (8).

Hyperparameters tunning Following the procedure presented in[2], we initialize P and Q as normally distributed random variables with small variance $\sigma^2 = 0.01$. We tuned both model parameters: the number of latent factors k and the regularisation penalty λ in the grid: $\lambda \in \{1, 5, 10, 15, 20\}$ and $k \in \{1, 5, 10, 20, 30, 40, 50, 60, 70, 80, 90, 100\}$.

Inductive Matrix Completion (IMC)

IMC[3] is a low-rank model that integrates drug and side effect attributes. For the binary matrix $X \in R^{n \times m}$ for n drugs and m side effects, let $K_d \in R^{n \times n}$ represent the kernel similarity matrix for drugs and, similarly, let $K_a \in R^{m \times m}$ represent the kernel similarity matrix for side effects. When only drug or side effect attributes are available, the kernel matrices are built using similarity measures. IMC aims to approximate X by:

$$X \simeq K_d W H K_a \tag{11}$$

By minimising the following loss function:

$$\min_{W,H} \mathcal{L}(W, H) = \frac{1}{2} \|\Omega \circ (X - K_d W H K_a)\|_F^2 + \frac{\lambda}{2} (\|W\|_F^2 + \|H\|_F^2) \tag{12}$$

Here, Ω represents a projection over the observed entries in X, $\circ$ is the element-wise product between matrices, $W \in R^{n \times k}$ is the drug latent representation matrix and $H \in R^{k \times m}$ is the side effect latent representation matrix. The second term in Eq. 12 is a $\mathcal{L}_2$ regularisation term applied to the latent matrices W and H, with a positive penalty $\lambda > 0$.

Gradient descent-based methods are required to approximate the solution. Thus, the derivatives of (12) are:

$$\frac{\partial \mathcal{L}(W, H)}{\partial W} = -K_d (X - \Omega \circ (K_d W H K_a)) K_a H^T + \lambda W \tag{13}$$

$$\frac{\partial \mathcal{L}(W, H)}{\partial H} = -W^T K_d (X - \Omega \circ (K_d W H K_a)) K_a + \lambda H \tag{14}$$

IMC uses chemical and drug target similarities for K_d and MedDRA taxonomic similarities for K_a. Following the procedure in[3], we used $K_d = \frac{1}{2}(A_{chem} + A_{DT})$ and $K_a = A_{MedDRA}$ as a combination model.

Optimisation We used conjugate gradient descent (CGD) and approximated line searches based on polynomial interpolation with Wolfe-Powel conditions to find the local minima in (12).

Hyperparameters tunning We initialize P and Q as normally distributed random variables with small variance $\sigma^2 = 0.01$. We tuned both model parameters: the number of latent factors k and the regularization penalty λ in the grid: $k \in \{1, 5, 10, 20, 30, 40, 50, 60, 70, 80, 90, 100\}$ and $\lambda \in \{1, 5, 10, 15, 20, 30, 40, 50\}$.

Feature-derived graph regularized matrix factorization (FGRMF)

The FGRMF[4] integrates several low-rank models, one per side information graph, by using a logistic regression model. Each low-rank model is optimised independently and then combined. For the binary matrix $X \in R^{n \times m}$ for n drugs and m side effects, FGRMF first minimises the following loss:

$$\min_{W,H} \mathcal{L}(W,H) = \underbrace{\frac{1}{2}\|X - WH\|_F^2}_{\text{low-rank model}} + \underbrace{\frac{\lambda}{2}(\|W\|_F^2 + \|H\|_F^2)}_{\text{regularisation}} + \underbrace{\frac{\alpha}{2}\|W\|_{\mathcal{D},\mathcal{G}}^2}_{\text{smoothness}} \tag{15}$$

where $\|.\|_F$ denotes the Frobenius norm, and $\|C\|_{\mathcal{D},\mathcal{G}}$ the Dirichlet norm on the graph $\mathcal{G} = (\{1,\ldots,n\},\mathcal{E},G)$, i.e. the weighted undirected graph with edge weights $g_{ij} > 0$ if $(i,j) \in \mathcal{E}$ and zero otherwise, representing the similarities between drugs.

The second step of FGRMF is to combined the solution from multiple graphs $\{\mathcal{G}_1, \mathcal{G}_2, ..., \mathcal{G}_p\}$, that gives different approximations to X, $\{\hat{X}_1, \hat{X}_2, ..., \hat{X}_p\}$ as follow:

$$h_\theta = \frac{1}{1 + \exp\left(-(\theta_0 + \sum_{i=1}^{p} \theta_i \hat{X}_i\right)} \tag{16}$$

where the weights θ_j are learned for each independent model. h_θ is used as a final prediction model.

Optimisation To find the solution in Eq. (15), we implemented the algorithm described by the authors. To optimise Eq. (16), we use the Matlab built-in function `mnrfit` (link function `logit`) to learn the coefficients estimates (θ_j).

Hyperparameters tunning We initialize W and H as normally distributed random variables with small variance $\sigma^2 = 0.01$. We tuned the three model parameters: the number of latent factors k, the regularisation penalty λ and the penalty α in the grid: $k \in \{1, 5, 10, 20, 30, 40, 50, 60, 70, 80, 90, 100\}$ and $\lambda \in \{1, 5, 10, 20\}$ and $\alpha \in \{0.01, 0.5, 1, 5\}$.

Geometric Self-Expressive Model (GSEM)

Optimisation We followed the recommended guidelines to implement non-negative matrix factorization (NMF)[5]. W and H were initialised with weights from a uniform distribution between $(0, \sqrt{\sigma}]$ for a $\sigma = 0.01$. The maximum number of iterations was set to 500 and `tolX` $= 0.01$. With this `tolX`, convergence occurs in roughly 50 iterations.

Hyperparameters tunning For each GSEM model, we tuned the parameters in the following grid: L2 regularisation parameter $a, c \in \{1, 10, 20, 30, 40, 50, 60, 70, 80, 90, 100\}$, L1 regularisation parameter $b, d \in \{0, 0.5, 1, 2, 3, 4, 5\}$, graph regularisation parameter $\mu, \alpha \in \{0.01, 0.1, 0.5, 1, 5, 10\}$. γ were set to 10^4 for all the experiments (we empirically observed that with this value diag(W)$\simeq 0$).

Computing infrastructure

All the models were run in a small cluster with 32 physical cores and 128GB of RAM. Each processor is a Intel(R) Xeon(R) CPU E5-2683 v4 @ 2.10GHz. We implemented most of the algorithms in Matlab R2019a 64-bit. Training of model hyperparameters was executed in parallel using the Parallel Computing Toolbox version 6.12.

Methods S2. Theoretical guarantees of GSEM. Related to STAR Methods.

Convexity

Here we prove that the GSEMW objective function is *convex*. The same procedure can be used to prove that the GSEMH objective is convex. Let us recall our objective function:

$$\mathcal{L}(W) = \underbrace{\frac{1}{2}\|X - XW\|_F^2}_{\text{self-representation}} + \underbrace{\frac{a}{2}\|W\|_F^2 + b\|W\|_1}_{\text{sparsity}} + \underbrace{\sum_i \frac{\mu_i}{2}\|W\|^2_{\mathcal{D},\mathcal{G}_i}}_{\text{smoothness}} + \underbrace{\gamma \operatorname{Tr}(W)}_{\text{null diagonal}} \tag{17}$$

Theorem 1 (Convexity) *For a real matrix X, the objective function $\mathcal{L}(W)$ is convex in the feasible region $W \geq 0$.*

We need to show that the Hessian of $\mathcal{L}(W)$ is a positive semi-definite (PSD) matrix. The Hessian of W is the second derivative of $\mathcal{L}(W)$, that is:

$$\nabla^2 \mathcal{L}(W) = X^T X + a + \sum_j \mu_j L_j$$

where $L_j = D_j - A_j$ is the graph Laplacian for the jth graph. The Hessian is PSD iif for a non-zero column vector $h \in R^m$ of real numbers the scalar $h^T \nabla^2 \mathcal{L}(W) h \geq 0$, i.e.,

$$\begin{aligned} h^T(X^T X + a + \sum_j \mu_j L_j)h \geq 0 \\ h^T X^T X h + a h^T h + \sum_j h^T \mu_j L_j h \geq 0 \\ \| Xh \|^2 + a \| h \|^2 + \sum_j \mu_j \| y_j \|^2 \geq 0 \quad \forall h, y, a > 0 \end{aligned} \tag{18}$$

In the last step, we use the fact that the Laplacian L_j is PSD and thus can be diagonalised as follow:

$$L_j = U_j^T \Lambda_j U_j = U_j^T \Lambda_j^{\frac{1}{2}} \Lambda_j^{\frac{1}{2}} U = (\Lambda_j^{\frac{1}{2}} U)^T (\Lambda_j^{\frac{1}{2}} U)$$

Letting $y_j = \Lambda_j^{\frac{1}{2}} U$, we see that:

$$h^T \alpha_j L_j h = \alpha_j y_j^T y_j = \alpha_j \| y_j \|^2 \geq 0$$

That is, the GSEMW objective function is convex in W.

Global optima convergence

The Karush-Khun-Tucker (KKT) complementary conditions of convergence are both necessary and sufficient conditions for a global solution point[6], given that the objective function $\mathcal{L}(W)$ is convex. We shall prove that our multiplicative learning algorithm satisfies the KKT conditions.

$$w_{ij} \leftarrow w_{ij} \frac{(X^T X + \sum_k \mu_k W A_k)_{ij}}{(X^T X W + \sum_k \mu_k W D_k + aW + b + \gamma I)_{ij}} \tag{19}$$

Theorem 2 (Convergence) *The cost function $\mathcal{L}(W)$ in Equation (17) converges to a global minimum under the multiplicative update rule in Eq. (19).*

KKT requires $w_{i,j} \geq 0$ and $(\nabla \mathcal{L}(W)_{ij} w_{ij} = 0$. The first condition holds with non-negative initialisation of W. For the second condition, the gradient is: $\nabla \mathcal{L}(W) = -X^T X - \sum_j \mu_k W A_k + X^T X W + \sum_k \mu_k W D_k + aW + b + \gamma I$, and according to the second KKT condition, at convergence $W = W^*$ we have $(X^T X W^* + \sum_k \mu_k W^* D_k + aW^* + b + \gamma I)_{ij} W^*_{ij} - (X^T X + \sum_k \mu_k W^* A_k)_{ij} W^*_{ij} = 0$, which is identical to (19). That is, the multiplicative rule converges to a global optima.

Methods S3. On the interpretability of our model vs NMF models. Related to STAR Methods.

In this supplementary section, we would like to comment on the differences between our matrix decomposition model proposed for predicting the frequencies of drug side effects[7] and GSEM.

The interpretability of our matrix decomposition model and GSEM are different because the models are different. First, we would like to clarify our MF model[7] addresses a different question (predicting frequencies of drug side effects), uses additional data (frequency of side effects), and does not integrate side information about drugs or side effects.

The model formulation can explain the difference in model interpretability between the two models. In our matrix decomposition model, each drug and each side effect are represented as low dimensional feature vectors such that their dot product encodes the frequency of the drug side effect. Let u_i denote the learned feature vector of drug i, and v_j the feature vector of side effect j. Then, the prediction score between drug i and side effect j is:

$$\text{MF score}_{ij} = u_i \cdot v_j$$

We have shown that each element in the learned vectors of the model can be related to anatomical categories of drugs and disorder categories of side effects. The interpretability capabilities of this MF model are to examine, for a new drug, the activation weights on each component of the learned vectors in relationship with their established biological effects.

Instead, GSEM learns "drug similarities" (as captured in H) and "side effect similarities" (as captured in W). For the side effect self-representation model, the score between a drug i and a side effect j can be written as follows:

$$\text{score}_{ij} = \sum_{k \in \{A,B,C\}} W_{kj}$$

Where $\{A, B, C\}$ is the set of known side effects induced by drug i. This simple formulation of our model allows for interpretability: the predicted drug-side effect association can be explained by a learned similarity between the known side effects induced by drug i and the side effect j. Side effects which are more similar to side effect j will have a higher impact in the overall score. In Figure 7 of the main manuscript, we have illustrated an example of the learned weights for two side effects: (i) vision blurred; and (ii) myocardial infarction.

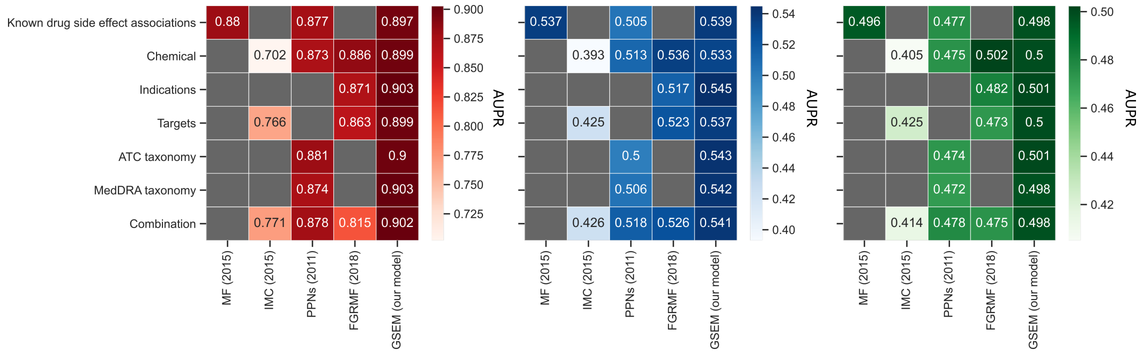

Fig. S1: Area Under the Precision Recall curve (AUPR) of each model type in each test set. Related to Figure 3. (red) Held-out test set containing clinical trials side effects; (blue) Post-marketing side effects from the SIDER database; (green) Postmarketing side effects from the OFFSIDES database.

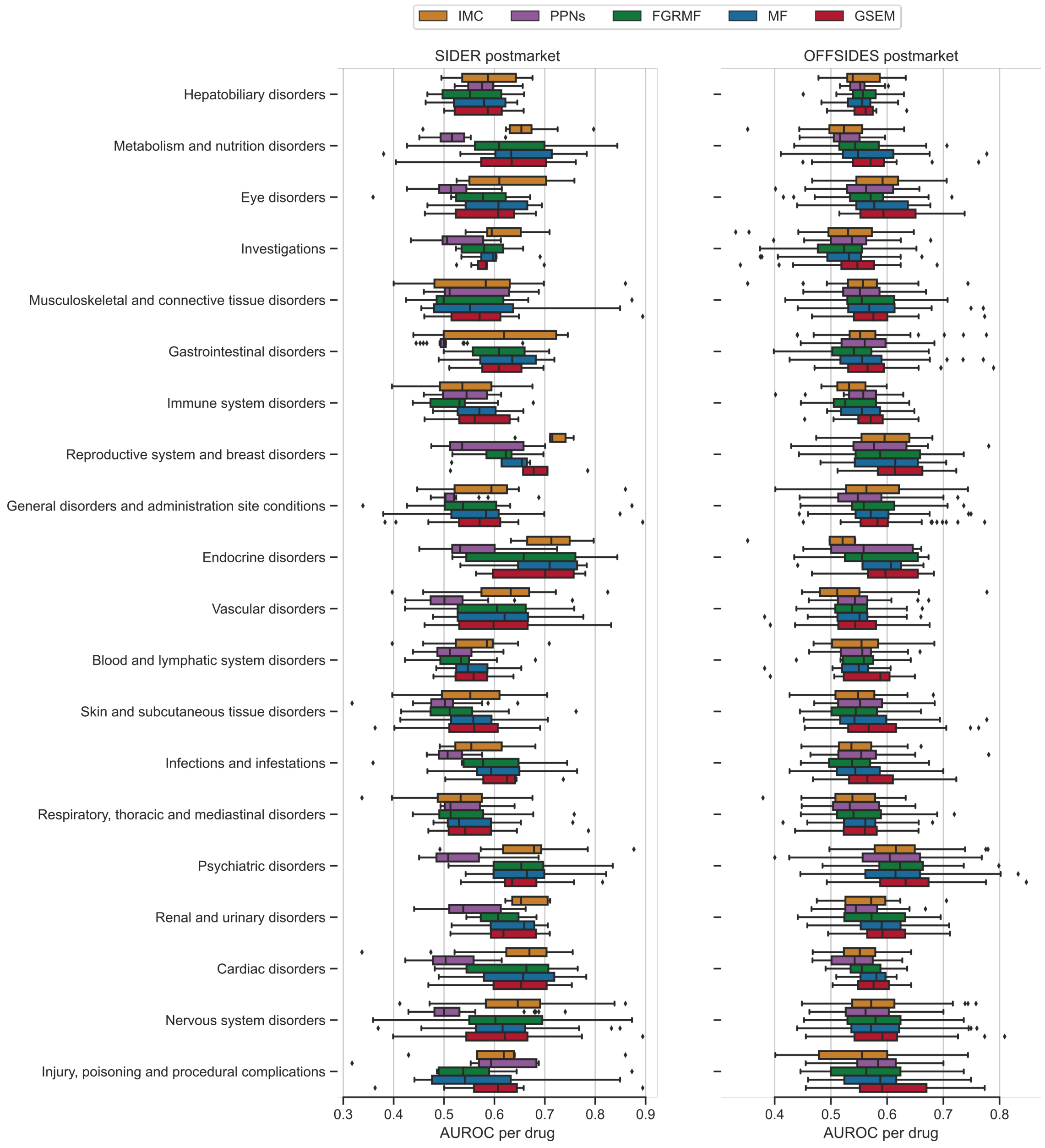

Fig. S2: Binary classification performance at predicting postmarket side effects in the SIDER and OFFSIDES databases. Related to Figure 3. AUROC computed for each drug by considering only the postmarketing side effects belonging to specific MedDRA category of disorders. (Left) SIDER postmarket set; (Right) OFFSIDES postmarket set.

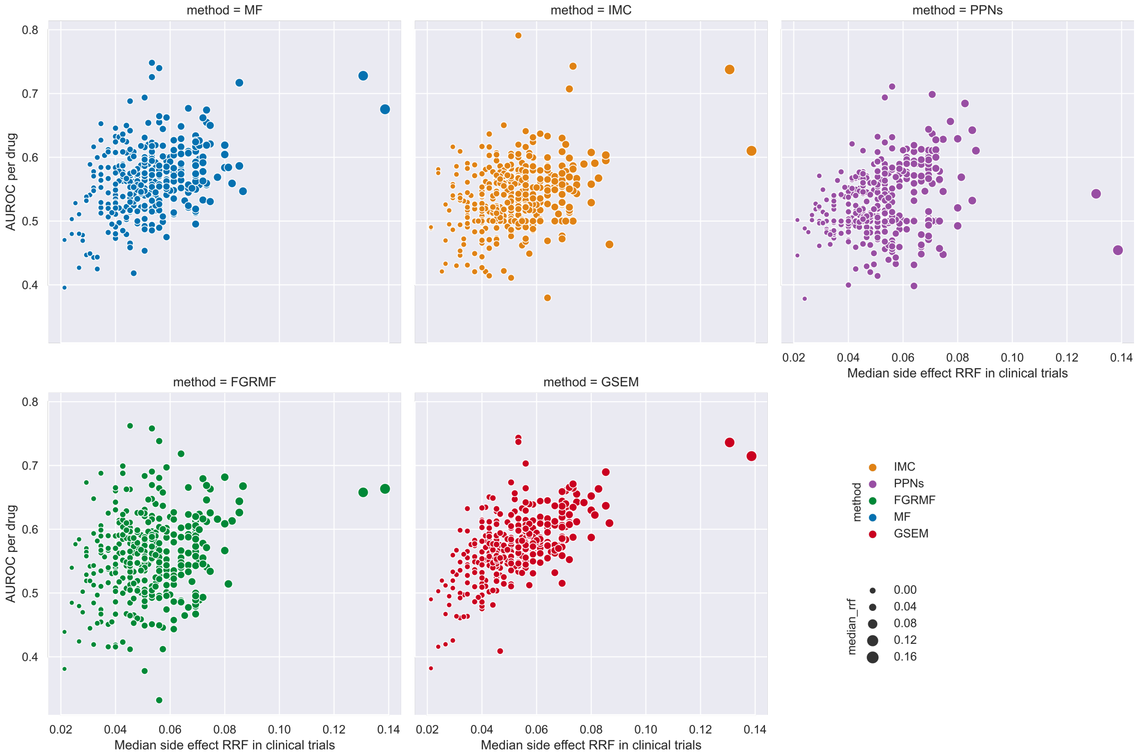

Fig. S3: Dependency between prediction performance and the median RRF values in the OFFSIDES postmarket test set. Related to Figure 5.

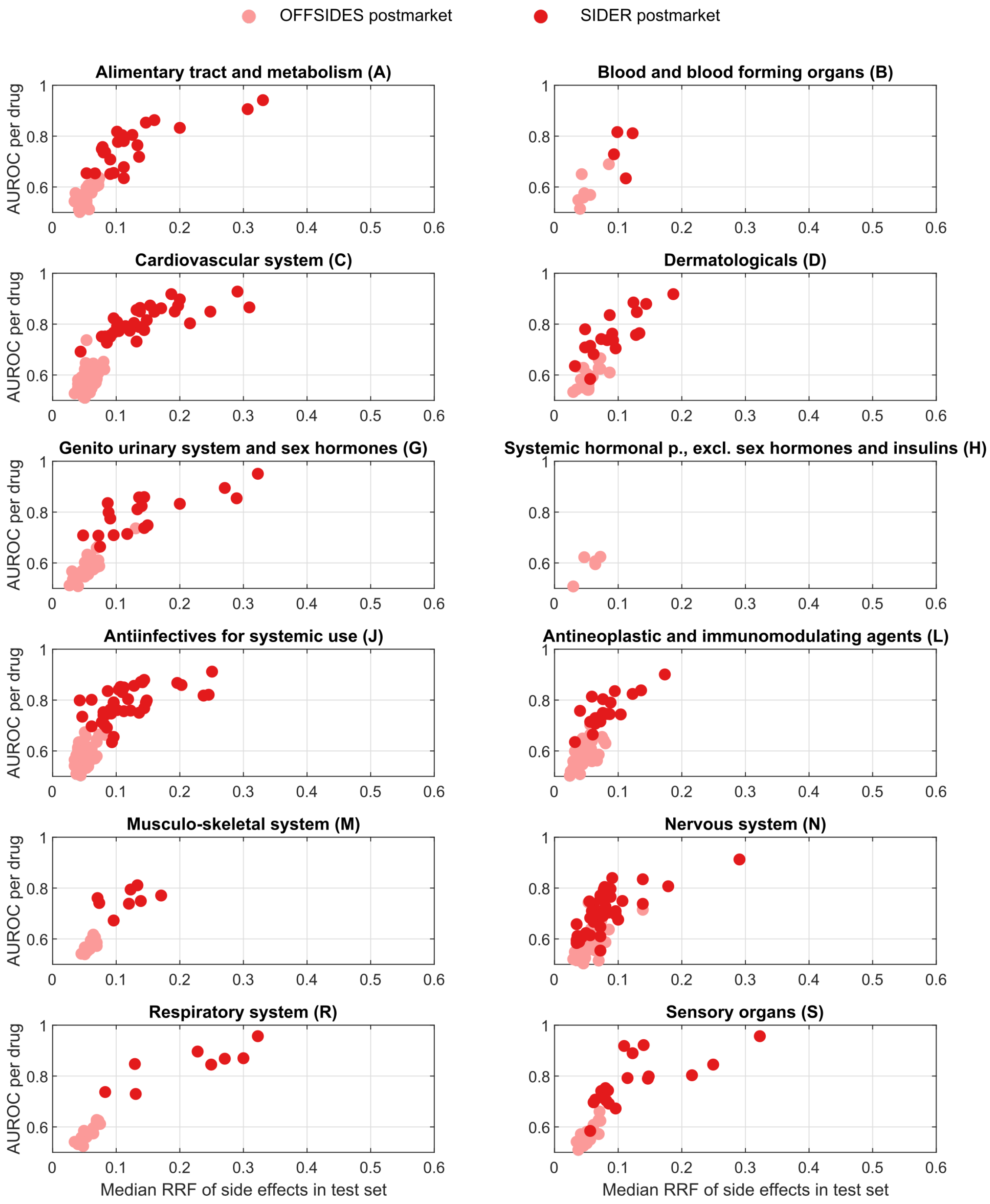

Fig. S4: Dependency between prediction performance for specific main drug Anatomical, Therapeutic and Chemical (ATC) categories and side effect RRF values in the postmarketing test sets. Related to Figure 5. Only drug categories with more than five drugs in the test sets are shown.

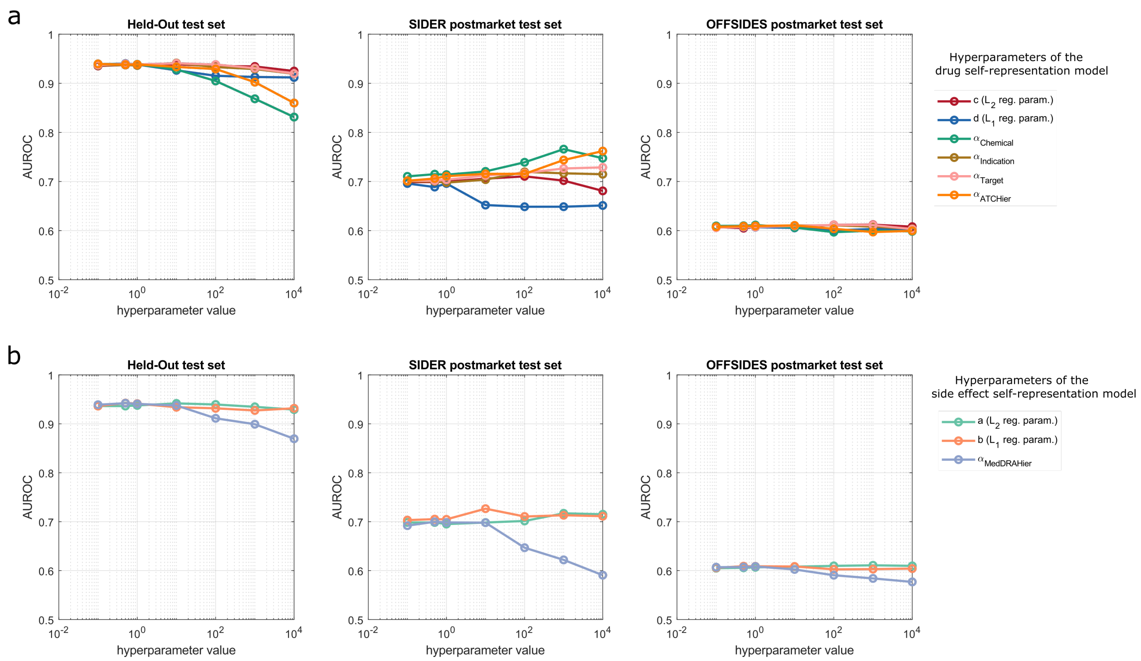

Fig. S5: Dependency between prediction performance and individual hyperparameters in GSEM. Related to Figure 3. (a) Drug self-representation model; (b) Side effect self-representation model. One hyperparameter at a time was change while all the others were set to zero.

References

Cami, A., Arnold, A., Manzi, S. and Reis, B. (2011), Predicting adverse drug events using pharmacological network models, Science translational medicine 3(114), 114ra127–114ra127.

Galeano, D. and Paccanaro, A. (2018), A recommender system approach for predicting drug side effects, in 2018 International Joint Conference on Neural Networks (IJCNN), IEEE, pp. 1–8.

Li, R., Dong, Y., Kuang, Q., Wu, Y., Li, Y., Zhu, M. and Li, M. (2015), Inductive matrix completion for predicting adverse drug reactions (adrs) integrating drug–target interactions, Chemometrics and Intelligent Laboratory Systems 144, 71–79.

Zhang, W., Liu, X., Chen, Y., Wu, W., Wang, W. and Li, X. (2018), Feature-derived graph regularized matrix factorization for predicting drug side effects, Neurocomputing 287, 154–162.

Berry, M. W., Browne, M., Langville, A. N., Pauca, V. P. and Plemmons, R. J. (2007), Algorithms and applications for approximate nonnegative matrix factorization, Computational statistics and data analysis 52(1), 155–173.

Boyd, S. and Vandenberghe, L. (2004), Convex optimization, Cambridge university press.

Galeano, D., Li, S., Gerstein, M. and Paccanaro, A. (2020), Predicting the frequencies of drug side effects, Nature communications 11(1), 1–14.
