# Supplementary material for: Machine learning prediction of side effects for drugs in clinical trials
Source: Cell Rep Methods. 2022 Dec 7;2(12):100358. doi: 10.1016/j.crmeth.2022.100358 (PMC9795366; doi:10.1016/j.crmeth.2022.100358)
Supplement: Document S2. Article plus supplemental information [file mmc2.pdf]

Cell Reports
Methods

Article

Machine learning prediction of side effects for drugs in clinical trials

Graphical abstract

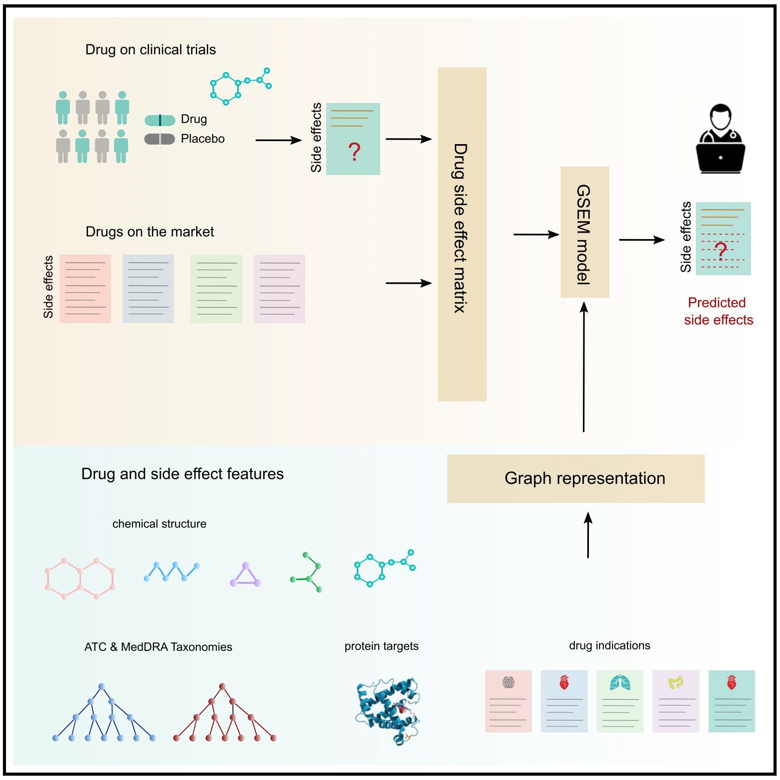

Authors

Diego Galeano, Alberto Paccanaro

Correspondence

dgaleano@ing.una.py

In brief

Oftentimes, side effects of drugs are discovered after the drug enters the market. What if scientists could better anticipate side effects during drug clinical trials? Galeano and Paccanaro develop an interpretable machine learning framework that can predict drug side effects based on the sparse information collected in drug clinical trials.

Highlights

- A machine learning framework for predicting side effects for drugs in clinical trials
- GSEM is a graph-based matrix completion model for heterogeneous data
- Side effects reported after a drug enters the market show a distribution shift
- A data integration technique to improve postmarketing side effect prediction

Galeano & Paccanaro, 2022, Cell Reports Methods *2*, 100358
December 19, 2022 © 2022 The Authors.
https://doi.org/10.1016/j.crmeth.2022.100358

CellPress

Cell Reports Methods

CellPress
OPEN ACCESS

Article

Machine learning prediction of side effects for drugs in clinical trials

Diego Galeano[1,4,*] and Alberto Paccanaro[2,3]
[1]Department of Electronics and Mechatronics Engineering, Facultad de Ingeniería, Universidad Nacional de Asunción, San Lorenzo, Paraguay
[2]School of Applied Mathematics, Fundação Getulio Vargas, Rio de Janeiro, Brazil
[3]Department of Computer Science, Centre for Systems and Synthetic Biology, Royal Holloway, University of London, Egham Hill, Egham, UK
[4]Lead contact
*Correspondence: dgaleano@ing.una.py
https://doi.org/10.1016/j.crmeth.2022.100358

MOTIVATION Drug side effects cause significant morbidity and mortality in healthcare. Side effects are discovered and added to the drug label during randomized controlled trials, but, due to trials' limited sample sizes, severe side effects are often discovered after the drug enters the market. An important question is whether we could use artificial intelligence to predict unknown side effects using the side effects identified during drug clinical trials. We studied this problem and developed a machine learning framework for predicting side effects for drugs undergoing clinical development.

SUMMARY

Early and accurate detection of side effects is critical for the clinical success of drugs under development. Here, we aim to predict unknown side effects for drugs with a small number of side effects identified in randomized controlled clinical trials. Our machine learning framework, the geometric self-expressive model (GSEM), learns globally optimal self-representations for drugs and side effects from pharmacological graph networks. We show the usefulness of the GSEM on 505 therapeutically diverse drugs and 904 side effects from multiple human physiological systems. Here, we also show a data integration strategy that could be adopted to improve the ability of side effect prediction models to identify unknown side effects that might only appear after the drug enters the market.

INTRODUCTION

Side effects of drugs are typically identified through randomized controlled clinical trials. It is well known that many side effects cannot be observed during clinical trials due to limitations in sample size and time frames. Postmarketing surveillance programs, such as the Adverse Event Reporting System (AERS), were designed to assist in the identification of side effects after the drug entered the market. However, the late identification of drug side effects is known to cause high morbidity and mortality in public healthcare,[1,2] the re-assessment of drug safety through new clinical trials,[3] and the possible withdrawal of drugs from the market.[4]

A wide range of computational approaches have been proposed to predict the side effects of drugs at different stages of the drug development process (see reviews by Ho et al.[5] and Boland et al.[6]). The first group of methods is applicable during pre-clinical drug development when only chemical, biological, and pharmacological information is available. These methods exploit chemical features,[7–11] protein targets,[12] and pathway information,[13] often in combination with protein networks,[14] and, in general, they offer a modest accuracy. A second group of methods was proposed for the postmarketing phase of drug development.[15–19] These methods exploit the side effects collected in clinical trials and the postmarketing phase to predict other unknown side effects. Our study differs from these methods in that we assumed that only side effects identified during clinical trials are available. This represents a more challenging scenario due to information sparsity and selection bias.[20,21] Our goal is 2-fold: (1) to simulate the realistic scenarios faced by safety professionals working in clinical drug development and (2) to provide a computational tool that can assist in the early detection of side effects of drugs undergoing clinical trials.

A critical application of our approach is during the different phases of clinical trials, where computational predictions can be used as a hypotheses generator to set the direction of the risk assessment. Our approach uses a matrix completion model that we called the geometric self-expressive model (GSEM). This is based on our objective function and multiplicative learning algorithm, which learns globally optimal solutions. Our model

© 2022 The Authors.
This is an open access article under the CC BY license (http://creativecommons.org/licenses/by/4.0/).

exploits known drug side effect associations and integrates graph structure information from chemical, biological, and pharmacological data. Here, we also show that predicting side effects that were identified after the drug entered the market from the information available during clinical trials is challenging. We attributed this to a distribution shift in side effect reports between clinical trials and postmarketing. This observation motivated a simple data integration technique that can be used to significantly improve the performance of GSEM at identifying side effects that might appear after the drug enters the market.

RESULTS

GSEM

Our starting point is the $n \times m$ drug side effect association matrix X, where $x_{ij} = 1$ if drug i is known to induce side effect j, or $x_{ij} = 0$ otherwise. Drugs can be related by their similarities in chemical structure, biological targets, and pharmacological activity. Side effects can also be related by their similarities in anatomical/physiological phenotypes. Our method integrates drug and side effect information by learning two similarity matrices: a drug similarity matrix $H \in \mathbb{R}^{n \times n}$ such that $X \simeq HX$ and a side effect similarity matrix W such that $X \simeq XW$. The GSEM generates scores for each drug-side effect pair by linearly combining these models:

$$\hat{X} = HX + XW. \quad \text{(Equation 1)}$$

The first term in Equation 1 is the drug self-representation model, and the second term is the side effect self-representation model. To learn W and H, we minimize the following objective functions:

$$\min_W \underbrace{\frac{1}{2}\|X - XW\|_F^2}_{self-representation} + \underbrace{\frac{a}{2}\|W\|_F^2 + b\|W\|_1}_{sparsity} + \underbrace{\sum_i \frac{\mu_i}{2}\|W\|_{\mathcal{D},\mathcal{G}_i}^2}_{smoothness} + \underbrace{\gamma Tr(W)}_{diagonal}$$

$$such\ that\ W \geq 0 \quad \text{(Equation 2)}$$

and

$$\min_H \underbrace{\frac{1}{2}\|X - HX\|_F^2}_{self-representation} + \underbrace{\frac{c}{2}\|H\|_F^2 + d\|H\|_1}_{sparsity} + \underbrace{\sum_j \frac{\alpha_j}{2}\|H\|_{\mathcal{D},\mathcal{G}_j}^2}_{smoothness} + \underbrace{\gamma Tr(H)}_{null\ diagonal}$$

$$such\ that\ H \geq 0 \quad \text{(Equation 3)}$$

where $\|.\|F$ denotes the Frobenius norm. We shall explain each term in Equation 2 only, as the same rationale can be applied to Equation 3. The first term in Equation 2 is the self-representation constraint, which aims at learning a self-representation matrix W such that XW is a good reconstruction of the original matrix X. The second term, in which $a, b > 0$ are constant values, is the sparsity constraint, which uses the elastic-net regularization known to impose sparsity and grouping effect.[22,23] The third term in Equation 2 is the smoothness constraint,[24–26] incorporating geometric structure into the self-representation matrix W from a given side effect similarity graph $\mathcal{G}_i$, with $\mathcal{G}_i = (\{1, ..., m\}, \mathcal{E}_i, A_i)$, i.e., the weighted undirected graph with edge weights $A_{ij} > 0$ if $(i,j) \in \mathcal{E}$ and zero otherwise. The smoothness constraint is important because it allow us to integrate into the model side information about side effects in the form of graphs. For a given side effect graph $\mathcal{G}$, the idea is that nearby points in $\mathcal{G}$ should have similar coefficients in W, which can be obtained by minimizing

$$\sum_{i,j} A_{ij}\|w_i - w_j\|^2 = Tr(WLW^T) := \|W\|_{\mathcal{D},\mathcal{G}}^2, \quad \text{(Equation 4)}$$

where w_i and w_j represent column vectors of W and $L = D - A$ is the graph Laplacian with $D = diag(\sum_j a_{ij})$. The constant values $\mu_i > 0$ in Equation 2 weigh the importance of the smoothness constraint for the prediction. When multiple graphs are combined, the parameters μ_i in Equation 2 tell us about the contribution and importance of the individual graph information for the prediction model. The fourth term in Equation 2 is a penalty for diagonal elements to prevent the trivial solution $W = I$ (the identity matrix). Typically, $\gamma \gg 0$ is used. The last constraint in Equation 2 is a non-negative constraint,[27] which is added here to favor interpretability of the learned W.

Figure 1 depicts an overview of our GSEM. The starting point is the matrix X containing binary associations encoding the presence or absence of drug side effects. The GSEM learns the self-representation matrices H and W that minimize our loss functions in Equation 3 and 2, respectively, by employing an iterative algorithm that uses a simple multiplicative update rule (see STAR Methods). Our algorithm is inspired by the diagonally rescaled principle of non-negative matrix factorization.[27] GSEM is fast to run, and it does not require setting a learning rate or applying a projection function. Our algorithm also satisfies global guarantees of convergence given by the Karush-Kuhn-Tucker (KKT) complementary conditions (proof in Methods S2). Having learned independently H and W, we calculate $\hat{X} = HX + XW$. Notice that while X contains binary values $[0, 1]$ that correspond to our original data, $\hat{X}$ contains real positive numbers that are our predicted scores.

Overview of evaluation

To obtain side effects identified in clinical trials, we followed the procedure in Galeano et al.[28] to retrieve side effects reported in randomized controlled studies from the Side Effect Resource (SIDER) 4.1.[21] 27,610 associations were obtained for n = 505 marketed drugs and $m = 904$ unique side effect terms. We also collected side effects identified after the drugs entered the market from two independent sources. 6,818 side effects reported in the postmarketing section of drug leaflets were obtained from the SIDER database (SIDER postmarket set). 25,797 statistically significant side effects reported in the AERS were obtained from the OFFSIDES database[29] (OFFSIDES postmarket set). The collection of drug side effect data used in our study is shown in Figure 2A.

Our goal is to assess the performance of the GSEM at predicting unknown side effects for drugs with a small number of side effects identified in clinical trials. Therefore, only side effects identified in clinical trials were used for training the model. Figure 2B illustrates how the clinical trials' side effects were randomly split into training, validation, and testing sets.

CellPress
OPEN ACCESS

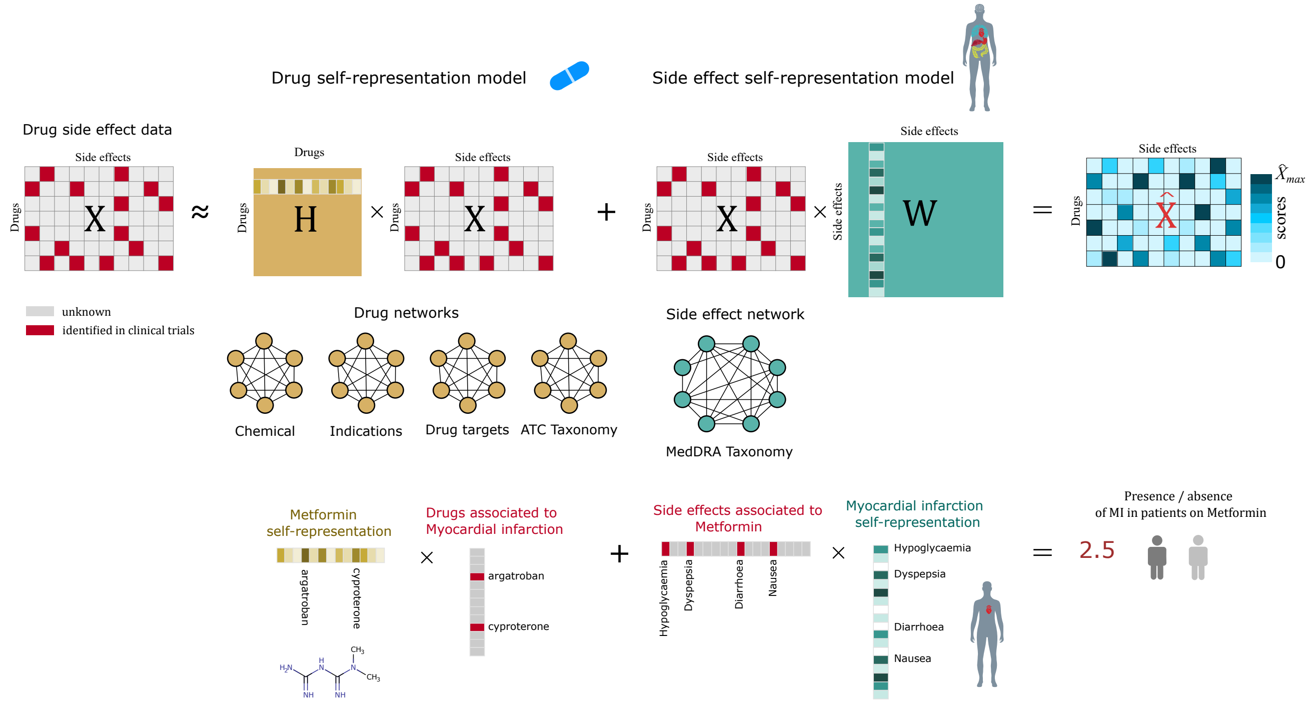

Figure 1. Geometric self-expressive model (GSEM)

27,610 associations identified on clinical trials for 505 drugs and 904 side effects were collected from the SIDER 4.1 database. The associations were arranged into an $n \times m$ matrix X by encoding their presence (= 1). Unknown associations were encoded with zeros (= 0). Our algorithm learns two similarity matrices that model the two pharmacological spaces of drug side effects. H (of size $n \times n$) encodes similarities between drugs that are learned from drug networks built from chemical, indication, target, and taxonomy similarities. W (of size $m \times m$) encodes similarities between side effects that are learned from physiological relationships between side effects. The GSEM learns independently H and W such that $X \simeq HX$ and $X \simeq XW$. By linearly combining these models, $HX + XW$, we obtain $\widehat{X}$, which models X, and where all the entries are replaced by real numbers—these are our predicted scores. Note that values replacing zero entries in X will constitute our predictions. Rows of H are drug self-representations, and columns of W are side effect self-representations. The lower illustration depicts how our model discovers a drug self-representation vector for the anti-diabetic drug metformin, and a self-representation vector for the side effect myocardial infarction (MI), such that the dot product of these vectors with the binary vector corresponding to known drugs for MI and known side effects of metformin, respectively, models the presence/absence of MI in patients on metformin. The body parts infographic vector was created by macrovector www.freepik.com.

Following previous approaches,[15–19] we framed our problem as a binary classification problem and used the area under the receiving operating curve (AUROC). The validation set consisted of 10% randomly held-out clinical trials side effects and randomly selected negatives of twice the number of positives. We used the validation set to tune the model hyperparameters. We then performed the evaluation by training the model with the combined training and validation sets using the optimal hyperparameters. We measure the AUROC and the area under the precision-recall curve (AUPR) on three test sets (see Figure 2C): (1) a held-out test set from randomly selected side effects identified in clinical trials, (2) postmarketing side effects from the SIDER database, and (3) postmarketing side effects from the OFFSIDES database.

We compared the prediction performance of the GSEM with a representative number of side effect prediction models that can also be applied to our problem: (1) matrix factorization (MF);[16] (2) predictive pharmacosafety networks (PPNs);[15] (3) inductive matrix completion (IMC);[17] and (4) feature graph-regularized MF (FGRMF).[18] Each side effect prediction model integrates different types of complementary information about drugs and side effects. We collected and used five types of side information for our study. For drugs, we obtained the chemical structure and protein targets from DrugBank,[30] indications from the Drug Repositioning Hub,[31] and Anatomical, Therapeutic, and Chemical (ATC) classification (see STAR Methods). We used MACCS fingerprints[32] to represent chemical structure and computed Tanimoto similarity using RDKit.[33] For side effects, we obtained the Medical Dictionary for Regulatory Activities (MedDRA) terminology. To build graphs from the different side information, we calculated the adjacency matrices using similarity measures (see STAR Methods). For the ATC and MedDRA terms, we also obtained their corresponding hierarchies to calculate taxonomy similarities that have been used by previous approaches.[15,17]

Evaluation of prediction performance on multiple drugs

Figure 3A shows the AUROC performance of the side effect prediction models at recovering missing drug-side effect associations in the held-out test set. Following a common practice in the literature,[15,17,18] we performed an ablation study. First, whenever possible, each method was trained using only the training matrix X without other side information (see first row in

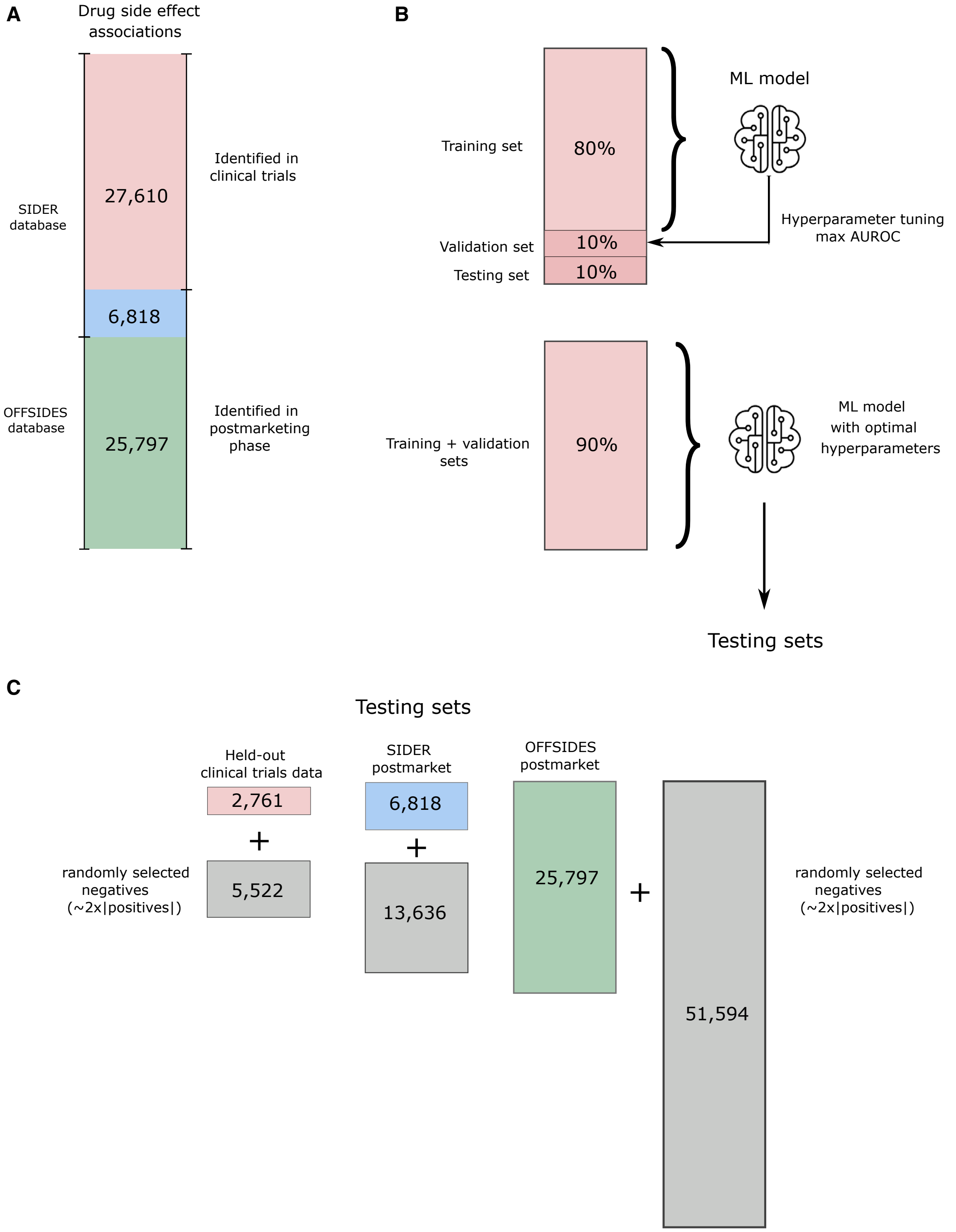

Figure 2. Evaluation procedure

(A) Drug side effect data were integrated from the SIDER 4.1 and OFFSIDES databases. They include a set of associations identified in clinical trials (red) and two sets of associations identified after the drugs entered the market: a postmarketing set from SIDER (blue) and OFFSIDES (green).

(B) The clinical trials association set was randomly split into training, validation, and test sets. Hyperparameters of each prediction model were tuned using the validation set. Each model was re-trained on the combined training and validation sets using optimal hyperparameters.

(C) Our test sets consisted of the held-out test set from the clinical trials set and the postmarketing sets from SIDER and OFFSIDES. Each positive set of associations was matched with a set of negatives twice their size, randomly selected.

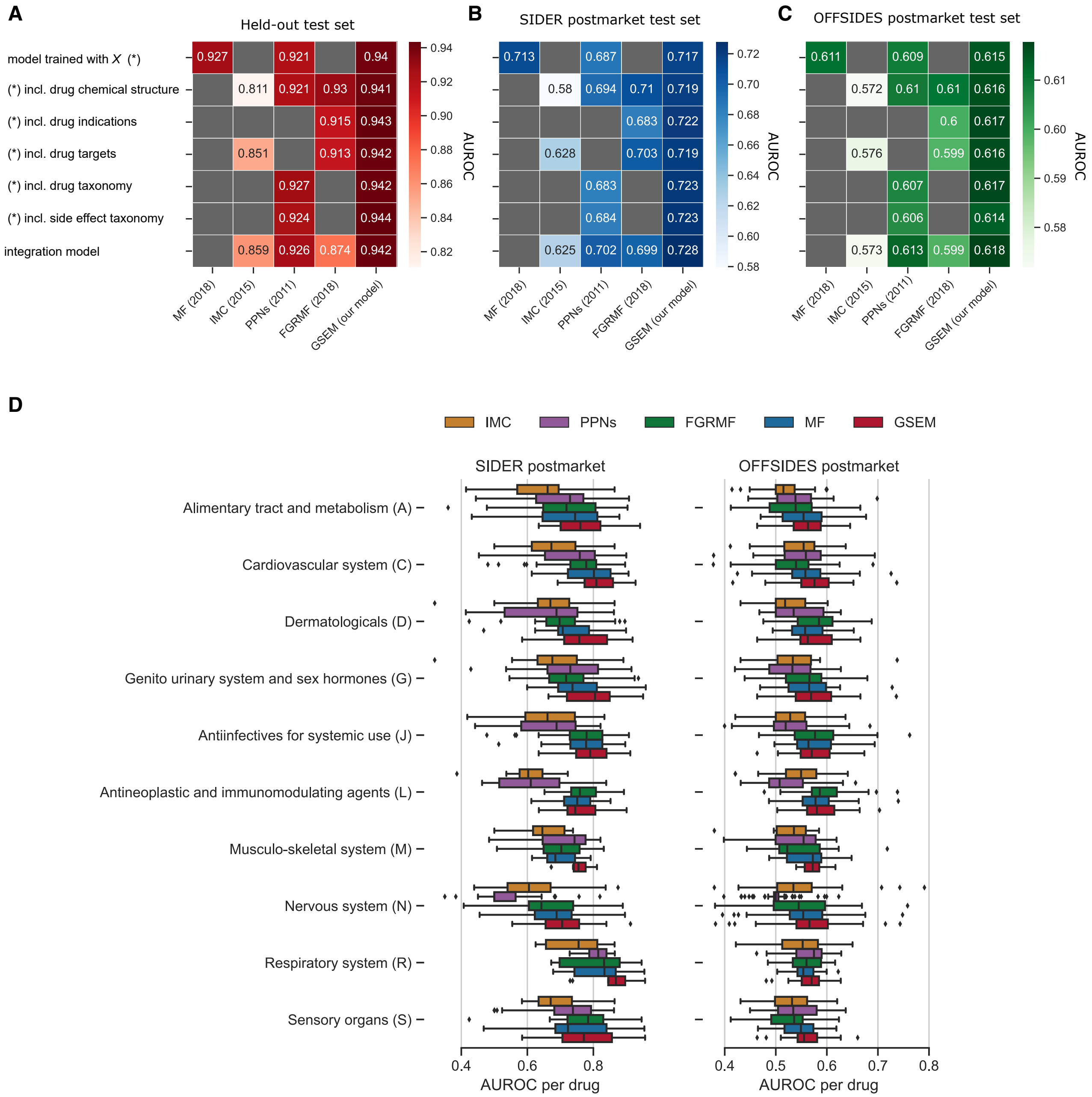

Figure 3. Performance evaluation on multiple drugs

Each model (x axis) was trained with drug side effect associations obtained from clinical trials, without other information (first row, y axis), or in combination with one side information type at a time (chemical, indication, target, and taxonomy similarities): second to fifth rows. The methods that proposed a model to integrate multiple side information are indicated as the integration model in the last row of the heatmap. Area under the receiver operating curve (AUROC) is shown only for the side information types used in the original publications of each competitor. Gray cells represent N/A. The binary classification performance is shown for three independent test sets.

(A) (Red) Held-out test set containing other clinical trials side effects.

(B) (Blue) Postmarketing side effects from the SIDER database, containing side effects reported in package inserts that were identified after the drugs entered the market.

(C) (Green) Postmarketing side effects from the OFFSIDES database, containing statistically significant side effects from the Adverse Event Reporting System (AERS) surveillance database.

(D) Drug-specific performance according to its main category according to the Anatomical, Therapeutic, and Chemical (ATC) classification. (Left) AUROC in the SIDER postmarket test set; (right) AUROC in the OFFSIDES postmarket test set.

Figure 3A). Second, if possible, one side information at a time together with X was integrated into the model to assess its contribution to the overall performance (second to fifth rows in Figure 3A). In these experiments, we run each method with the side information types proposed in the original publications (see Methods S1). Finally, if the original publications proposed a way to integrate multiple information types (more than one) in their framework, we implemented them, and their performance is shown in the last row of Figure 3A. Notice that the GSEM, as proposed in Equations 3 and 2, is a model that allows for the integration of multiple types of heterogeneous information.

On the held-out test set with other side effects identified in clinical trials, the GSEM outperforms all the competitors by 1.4%–13.3%. Even when training GSEM using the training matrix X alone, i.e., without side information, the GSEM achieves 0.940 in terms of the AUROC. This baseline performance can be slightly improved using side information for drugs and side effects. Other methods, such as PPNs[15] and IMC,[17] also show a similar trend; therefore, side information should be used when available. In addition, we observed that while the competitors' performance is more sensitive to the specific choice of side information, the performance of the GSEM displays a small variability across information types. The mean and SD AUROCs in the held-out test set are 0.9421 ± 0.0012 (GSEM) versus 0.9079 ± 0.0207 (FGRMF), 0.8405 ± 0.0026 (IMC), and 0.9239 ± 0.0212 (PPNs). GSEM also consistently outperforms the competitors in terms of the AUPR (Figure S1).

We then tested our method in a more realistic scenario using a simulated prospective evaluation similar to the one used by Cami et al.[15] In this procedure, all side effects identified after the drugs entered the market were used as a test set (postmarket test sets in Figure 2B). Figures 3B and 3C show the prediction performance of the methods in postmarketing test sets. The GSEM outperforms the competitors by 1.5%–14.8% in the SIDER postmarket test set and by 0.7%–4.6% in the OFFSIDES postmarket test set.

Interestingly, the GSEM offers the best prediction performance in both prospective sets when combining all available side information. Following Cami et al.,[15] we further asked whether the performance of the models varies for drug- or side effect-specific categories. We performed a second evaluation where we used the best-performing models of each column of Figure 3A to analyze the performance of a specific group of drugs and side effects (see STAR Methods). Figure 3D shows the AUROC performance of the models for drug-specific anatomical categories according to their primary ATC classification. For most categories, the GSEM's mean AUROC was above 0.75 in the SIDER postmarket test set—we obtained the lowest AUROC performance for nervous system drugs (0.706) and the highest performance for respiratory system drugs (0.852). In the OFFSIDES test set, the mean AUROC was above 0.55 for all the categories. The performance of the models for the side effect-specific MedDRA category of disorders are shown in Figure S2.

Distribution shifts in side effects reported before and after the drugs enter the market

An important observation from Figures 3A–3C is that there is a considerable difference in AUROC performance when predicting other side effects from clinical trials (GSEM AUROC of 0.944) versus postmarketing (GSEM AUROCs of 0.728 and 0.618 in the SIDER and OFFSIDES postmarket sets, respectively). These differences cannot be explained by the specific method used or the type of side information used in the integration. The differences in prediction performance prompted us to ask whether they can be explained by a distribution shift in side effect reports before and after the drug enters the market.

To analyze differences in reporting trends, we defined the ratio of reporting frequency (RRF) as the normalized count of drugs associated with a given side effect (see STAR Methods). The RRF reflects whether a side effect has been associated with many or few drugs in our dataset. For instance, nausea, a side effect reported on most drugs, has an RRF of 1.0, while eye infection, reported only on a few drugs, has an RRF of 0.011. We contrasted the RRF of each side effect computed using clinical trial associations versus postmarketing associations. Figures 4A and 4B show that side effects reported in clinical trials and postmarketing follow a different trend. A side effect reported on a small number of drugs in clinical trials (low RRF in the x axis) can be reported on many drugs in the postmarketing phase. This trend is even more prominent in the OFFSIDES postmarket set. For comparison, the expected trend without distribution shift is shown in Figure 4C for a held-out set from clinical trials associations (Pearson, $\rho = 0.923$, $p < 2.23 \times 10^{-308}$). Our results suggest differences in reporting trends between drug side effect associations reported in clinical trials and the postmarketing phase.

We further explored whether there are statistically significant differences in RRF values for drug anatomical classes and side effect disorder types. We grouped drugs by their main ATC classification and compared distributions of RRF values based on the known side effects reported in different sets (see STAR Methods). Figure 4D shows that for the majority of drug categories, the side effects that were reported in clinical trials tend to be biased toward frequently reported side effects except for nervous system drugs. Conversely, while the SIDER postmarket set tends to be more significant toward rarely reported side effects in clinical trials, the OFFSIDES set was more significant for frequently reported side effects. We repeated our statistical analysis by grouping side effects based on their main MedDRA category of disorders. Figure 4E shows that side effect categories are significant toward rarely reported side effects, i.e., low RRF values.

A fundamental assumption in machine learning is that the training and testing sets are drawn from the same underlying distribution.[34] Our analysis in Figure 4 shows that this is not the case for our problem. We hypothesized that the distribution shifts in side effect reports between clinical trials and postmarketing could explain the differences in prediction performance that we observed in Figures 3A–3C. It would imply a dependency between the AUROC performance and the RRF values of the side effects in the test set. To explore this dependency in more detail, we calculated AUROC values for single drugs on the SIDER postmarket test set. Figure 5 shows a correlation between prediction performance and the RRF values of the side effects we are trying to predict. A positive correlation is observed for all the methods, suggesting that each drug's

CellPress
OPEN ACCESS

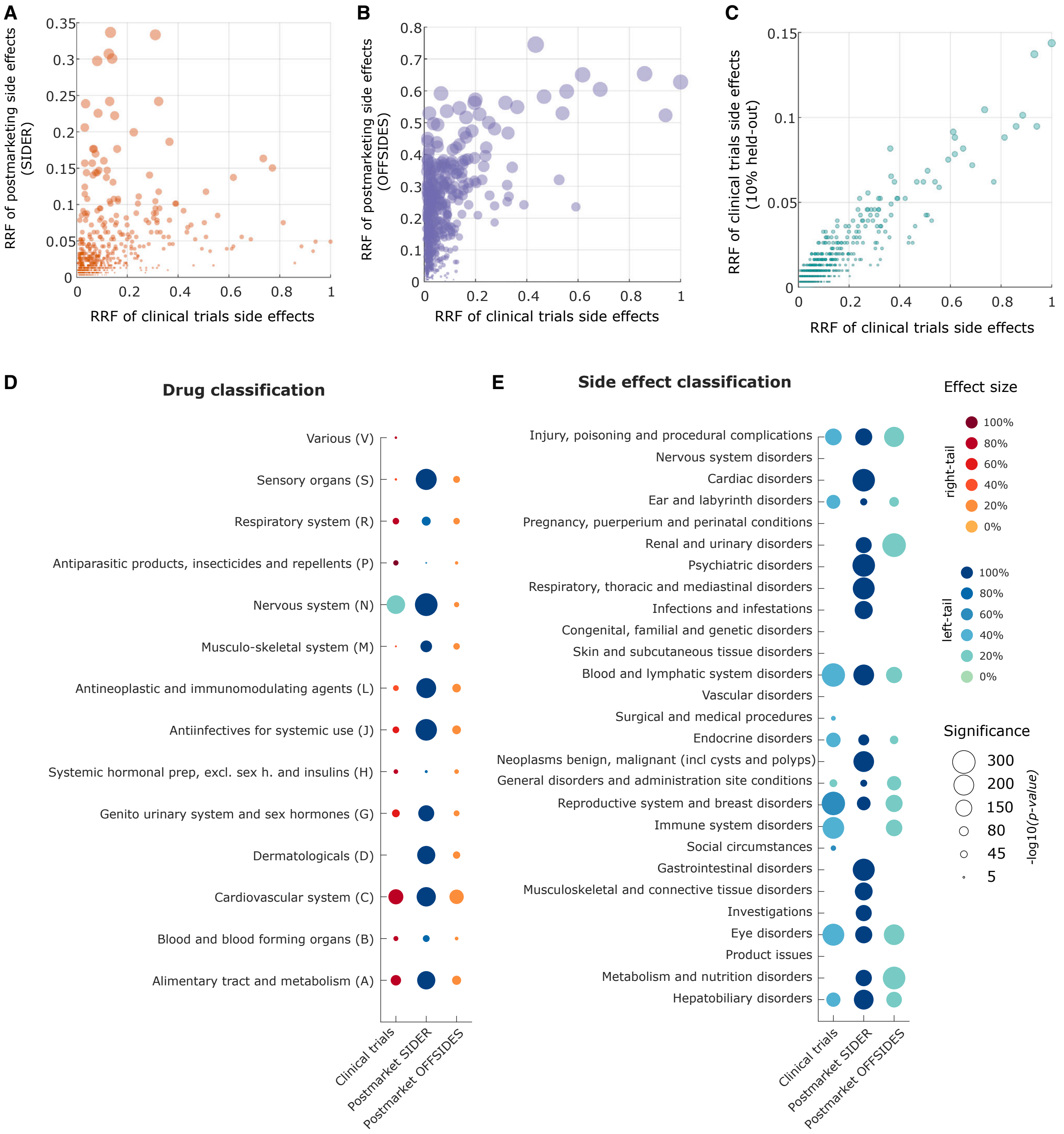

Figure 4. Differences in the distribution of side effect reports in clinical trials and postmarketing drug development phases

Side effect ratio of reporting frequency (RRF) is a normalized count of drugs associated with a given side effect. Each point represents a side effect, and the RRF values of side effects identified in clinical trials are compared against (A) the RRF of of side effects identified in postmarketing as found in the SIDER database (Pearson, $\rho = 0.377$, $p < 5.1 \times 10^{-3.2}$); (B) the RRF of side effects identified in postmarketing as found in the OFFSIDES database (Pearson, $\rho = 0.192$, $p < 6.4 \times 10^{-9}$); and (C) the a held-out set (Pearson, $\rho = 0.923$, $p < 2.23 \times 10^{-308}$). The size of the circle is proportional to the RRF values.

(D and E) Statistical analysis of side effect RRF significance for (D) ATC group of drugs and (E) MedDRA-group of side effects. Only statistically significant associations are shown (one-tailed Wilcoxon rank-sum test with Benjamini-Hochberg adjusted significance, $p < 0.05$). The circle size represents the significance (p value), and the color encodes the effect size of the association—the difference between the median in the group compared with the median of all drugs (or side effects). Colors separated the effect size to indicate whether the one-tailed significance was right-tailed (red palette) or left-tailed (blue palette).

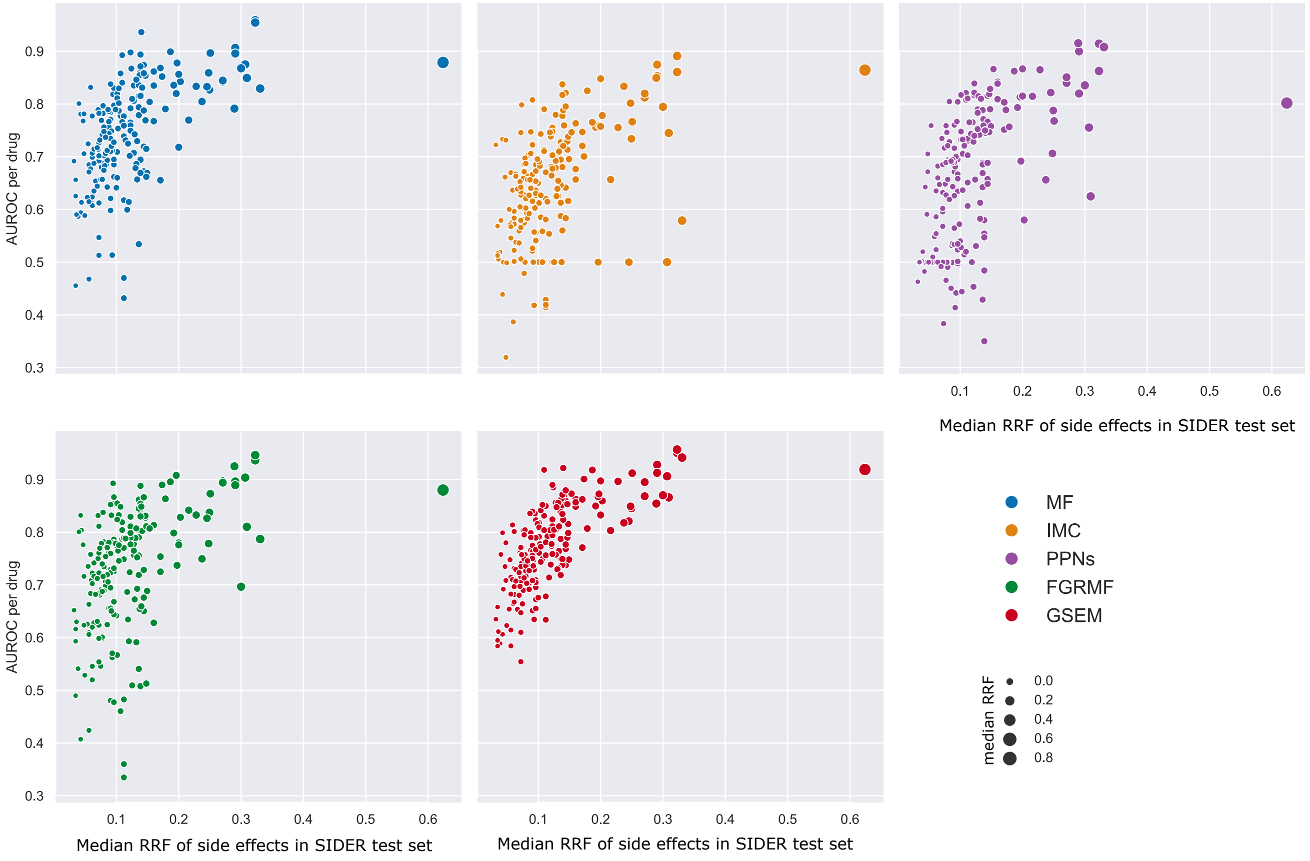

Figure 5. Dependency between prediction performance and side effect RRF value

Each model generated scores by training with clinical trials' side effects and side information. Models were then assessed, for each drug, in their ability to identify the presence or absence of postmarketing side effects (SIDER postmarket test set) out of all the unknown side effects for the drug. Each dot in the figure represents an individual drug. The performance per drug is shown in the AUROC (y axis) versus the median RRF of the side effects in the test set (x axis). There is a direct correlation between the prediction performance of the each model and the median RRF value of the side effects in the test set: MF (Pearson correlation, $\rho = 0.53$, $p < 3.54 \times 10^{-16}$); IMC ($\rho = 0.51$, $p < 1.40 \times 10^{-14}$); PPNs ($\rho = 0.55$, $p < 2.85 \times 10^{-17}$); FGRMF ($\rho = 0.45$, $p < 2.50 \times 10^{-11}$); and GSEM ($\rho = 0.68$, $p < 4.11 \times 10^{-28}$). Each point represents a drug, and the circle's size is proportional to the median RRF.

prediction performance depends on the magnitude of the distribution shift.

Reported side effects in OFFSIDES have even lower RRF values than those in SIDER (see Figure S3), thus explaining the differences in AUROC performance between SIDER and OFFSIDES postmarket sets in Figures 3B and 3C, and Figure S4 shows that the AUROC per drug varies by category depending on the RRF values of the side effects in the postmarketing test sets.

A data integration technique to improve prediction performance

We propose a simple data integration technique to improve the prediction performance of side effect prediction models for individual drugs. Our idea is based on the observation that the effect of the distribution shift can be reduced if we integrate postmarketing data into the training matrix X. Figure 6B shows that the RRF values of specific side effects can be improved using postmarketing information in training.

Figure 6A illustrates our evaluation procedure for single drugs. For a given drug x, we used its clinical trials side effects for training and its combined SIDER and OFFSIDES postmarketing side effects for testing. Then, we assessed the AUROC performance using two strategies that differ in the information used for the other drugs. The first strategy uses only side effect associations reported in clinical trials. The second strategy uses side effect associations reported in clinical trials and postmarketing. To prevent data leakage, we removed other chemically similar drugs from the training matrix X (see STAR Methods). Notice that for both strategies, we trained each method using the same set of optimal hyperparameters obtained in the validation set, as shown in Figure 2.

Figures 6B and 6C shows the AUROC performance of the side effect prediction models using strategies 1 and 2. The inclusion of the postmarketing side effects for the other drugs used for training dramatically affected the prediction performance for single drugs. The mean AUROC improved from 0.604 to 0.667 for MF; 0.512 to 0.537 for IMC; 0.596 to 0.650 for FGRMF; 0.60 to 0.733 for PNN; and 0.616 to 0.746 for the GSEM. Our method shows a 13% performance improvement using strategy 2.

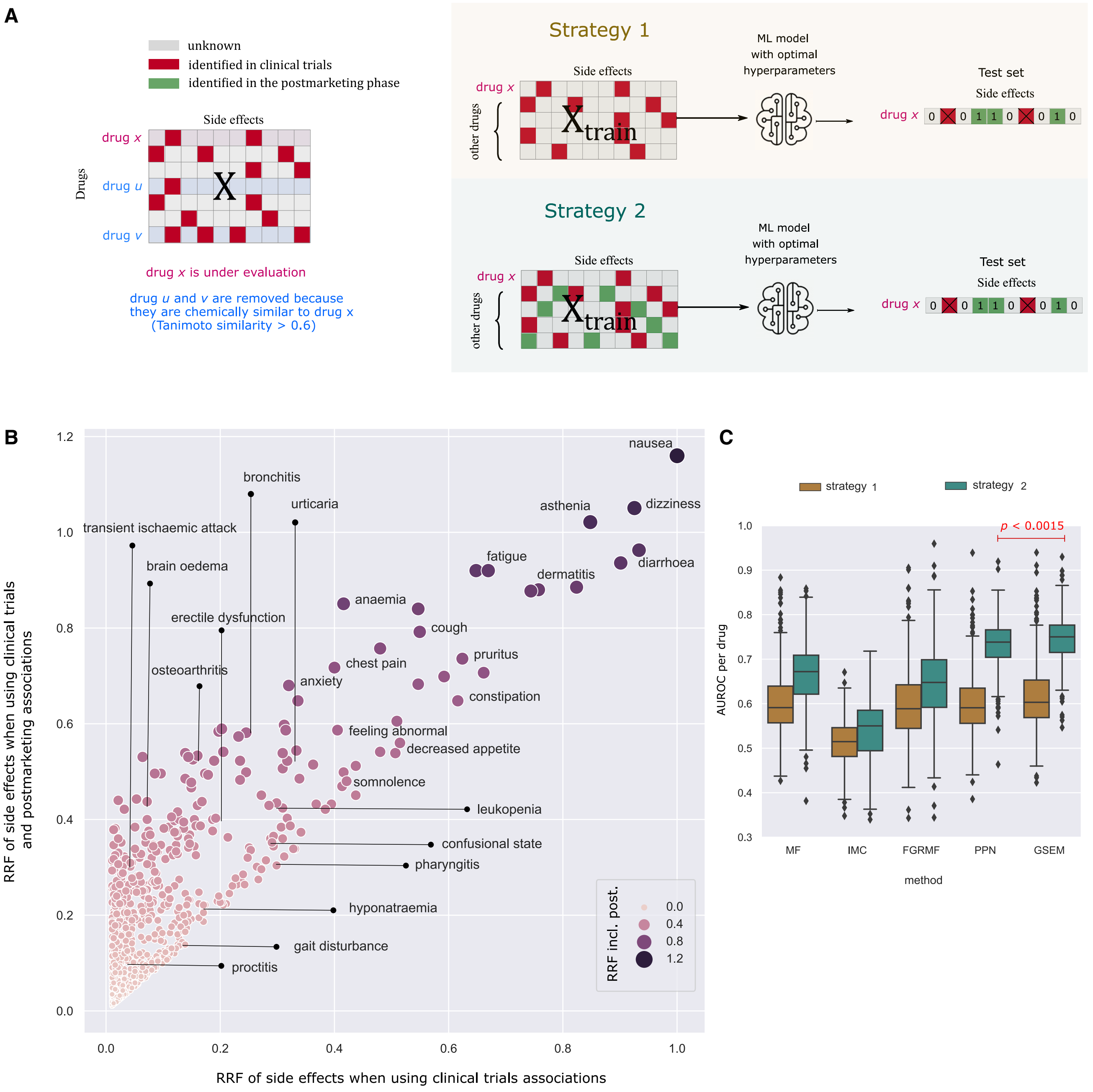

Figure 6. A data integration strategy for predicting postmarketing side effects for drugs in clinical trials

(A) Evaluation procedure for single drugs to predict side effects identified after the drugs enter the market (postmarketing) using for training side effects identified in clinical trials. For a given drug *x*, we performed two evaluation strategies that change the set of associations used for the other drugs in *X*: (1) uses only clinical trials side effects and (2) uses clinical trials and postmarketing side effects. Side effects chemically similar to drug *x* were removed from the training matrix to avoid data leakage (illustrated as drugs *u* and *v*).

(B) Comparison of side effect RRF values when using only clinical trials associations (x axis) and when also including also the postmarketing associations (y axis). Each point represents a side effect, and the circle's size is proportional to the RRF when including postmarketing side effects. Several side effect terms are indicated.

(C) Boxplots of the AUROC per drug on the combined postmarketing test sets using strategies 1 and 2. The distribution of AUROC values for the GSEM using strategy 2 is significantly better than that of the best competitor (PPN) (one-tailed Wilcoxon rank-sum test $p < 0.0015$).

Self-representations capture biological relationships

Two properties make the GSEM an interpretable and reproducible model. First, the GSEM is interpretable because the predicted score can be explained in terms of learned similarities between drugs and side effects. Second, the GSEM's solutions are reproducible because the learned solution is a globally optimal solution of its objective function. The GSEM overcomes the common problem of machine learning models that learn different solutions even when training the same model with a different random initialization, which is persistent in deep-learning models.[35]

The GSEM's predicted score for a drug *i* and side effect *j* can be written as follows:

$$\widehat{X}_{ij} = \sum_{\substack{u \in \text{drugs known to} \\ \text{cause side effect } j}} H_{iu} + \sum_{\substack{v \in \text{side effects caused} \\ \text{by drug } i}} W_{vj}, \quad \text{(Equation 5)}$$

where H and W are non-negative. The first term in Equation 5 contains the learned similarities between drug i and the drugs known to cause side effect j. The second term in Equation 5 contains the learned similarities between side effect j and the side effects known to be caused by drug i. If any of the individual terms in the sum is high, the prediction score $\widehat{X}_{ij}$ will be high because the model allows only for summation and not the subtraction of terms.

We hypothesized that the learned H can capture biological relationships between drugs. Following a similar procedure to Cheng et al.,[36] we assessed whether our drug similarity measure, defined as $(H + H^T)/2$ (see STAR Methods), reflects known chemical, biological, and pharmacological relationships between drugs. To be sure that there is no information leakage, we trained the GSEM using all available clinical trials and postmarketing information (encoded in X) but without any side information (i.e., $\mu_i = \alpha_j = 0 \forall i, j$) (see STAR Methods). We found that our drug similarity based on H correlates with chemical, indication, target, and ATC taxonomy similarities (Figure 7B). Interestingly, our drug similarity was also indicative that the drugs were pharmacologically similar (ATC taxonomy similarity above 0.05) or distinct (below 0.05). Our results suggest that the matrix H in our model could capture chemical, biological, and pharmacological relationships between drugs.

We also tested whether W could capture the anatomical/physiological relationships between side effect phenotypes, as defined by the MedDRA taxonomy similarity (see STAR Methods). We defined side effect similarities based on W as $(W + W^T)/2$ (see STAR Methods). We found that the side effect similarities based on W correlate with the MedDRA taxonomy similarity (Figure 7B, bottom). We observed that phenotypically similar side effects tend to have similar self-representations. The similarity also indicates whether side effects are anatomically/physiologically similar (MedDRA taxonomy similarity above 0.05) or distinct (below 0.05).

To showcase how the learned matrices allow for interpretability, we explored the weights in W for two side effects: (1) myocardial infarction (MI), which has been associated with the withdrawal of many drugs from the market,[4] and (2) blurred vision. Figure 7A shows a diagram of the side effects that are more similar to MI and blurred vision based on the weights in W. We observed that MI is very similar to other vascular-related disorders, including angina pectoris, which has been shown to appear prior to MI.[37] 46 drugs in our dataset are known to be associated with both angina pectoris and MI, which might explain the learned association. On the other hand, blurred vision, which is classified in MedDRA as both an eyes and nervous system disorder, is also very similar to other related conditions, including psychiatric disorders. The learned matrix W allows for a transparent inspection of how the model arrived at a given prediction. If a drug is known to induce MI, our model predicts that the drug might also induce similar side effects, as shown in Figure 7A.

DISCUSSION

Here, we introduced the GSEM, a computational approach for predicting the side effects of drugs during clinical drug development. Instead of waiting for postmarketing observational evidence to be accumulated, our framework can be used to assist drug safety professionals in the identification of side effects during drug clinical trials. To show this, we trained the models with side effects identified in clinical trials and tested them to predict side effects identified in the postmarketing phase. To our knowledge, this is the first attempt to predict the presence or absence of side effects for drugs with a small number of side effects identified in clinical trials. Our framework can be used together with our recent approach to predict the frequencies of drug side effects in patients.[28] These tools can be helpful in the early detection of rare side effects that cannot be effectively captured in small-size clinical trials.

Our analysis indicated that predicting side effects that were identified after the drugs entered the market is difficult when training only with side effects identified during clinical trials. Part of this difficulty lies in the differences in the distribution of side effects reported in clinical trials and in postmarketing. Scarcely reported side effects during clinical trials tend to be highly reported in postmarketing, thus explaining the models' difficulty at predicting them. We further studied this issue by analyzing the dependency between the number of drugs associated with a side effect (RRF value) and the prediction performance of machine learning models (see Figure 5). Our experiments showed that the prediction performance of the models heavily depended on the RRF value of the side effects we were aiming to predict. Strikingly, improving the RRF value of each side effect by adding information from postmarketing reports was more critical for improving the prediction of postmarketing side effects than the use of any drug or side effect features.

The problem of distribution shift in side effect reports is deeply connected to the intrinsic distributional properties of drug side effects. In a previous study,[28] we have shown that drug side effect reports follow a long-tailed distribution. The distribution can be summarized in a Pareto 80/30 rule, where 80% of the associations come from 30% of the side effects.[28] Unfortunately, this means that the amount of labeled information (captured by RRF), vital for machine learning models, varies per side effect, following an almost exponential distribution. It would be essential to consider the dependency between prediction performance and side effect RRF when evaluating computational models that aim to predict drug side effects.

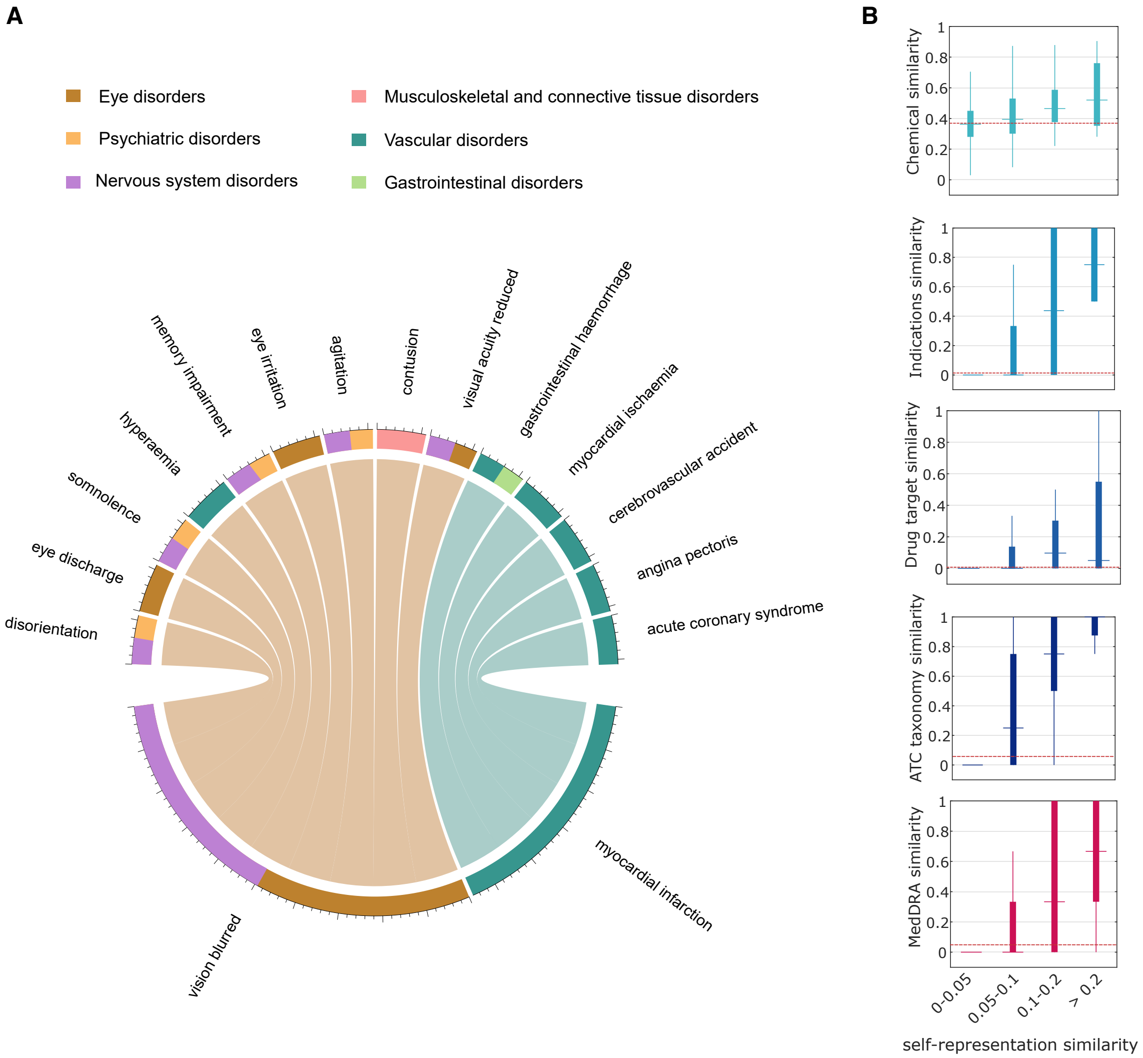

Figure 7. Self-representations capture chemical, biological, and pharmacological relationships

(A) Diagram representing how vision blurred and MI (bottom) are self-represented with other side effects (top). Only side effects with self-representations weights above 0.05 are shown. The thickness of the connections is proportional to the self-representation weights in *W*. The colors in the outer circle represent the disorder category of the side effect according to the Medical Dictionary for Regulatory Activities (MedDRA) terminology.

(B) The interplay between the drug self-representation similarity and four types of drug-drug similarities: chemical, indications, target, and ATC taxonomy. The bottom figure shows the interplay between the side effect self-representation similarity and the MedDRA taxonomy similarity. Mean values of chemical (mean similarity of 0.3689), indications (0.0134), drug target (0.0076), ATC taxonomy (0.0576), and MedDRA taxonomy (0.0488) similarities are shown as dashed horizontal lines.

An innovative aspect of our algorithm is that it learns similarities between drugs (matrix *H*) and between side effects (matrix *W*). Our model is fundamentally different from previous side effect prediction models. A PPN[15] is a network-based method that builds topological features from the bipartite drug-side effect graph. The graph is obtained when connecting the nodes representing drugs to the set of nodes representing side effects. PPNs also integrate chemical, taxonomic, and biological features and then use a logistic regression model to predict. MF[16] is a matrix decomposition model that learns a low-dimensional feature vector for each drug and a low-dimensional feature vector for each side effect such that the dot product between the vectors models an entry in *X*. It amounts to a low-rank approximation of *X*. Similarly, FGRMF[18] uses several low-rank approximation models for each drug side information graph that are integrated into the model using the smoothness constraint.[24–26] The final FGRMF score is the probability given the logistic regression that combines the scores of the individual low-rank models. Finally, IMC[17] is an IMC model that integrates drugs and side effect features in the matrix decomposition model. A detailed description of the mathematical formulation of each competitor, together with their implementation and optimization, can be found in Methods S1.

GSEM builds upon the recent development of high-rank matrix completion based on self-expressive models (SEM)[38] and sparse

linear methods,[39] as well as the recent trend of deep learning on graphs.[26,40,41] SEMs represent data points, e.g., drugs, approximately as a linear combination of a few other data points. Elhamifar[38] proposed SEMs as a framework for simultaneously clustering and completing high-dimensional data lying in the union of low-dimensional subspaces. It has been shown that SEMs generalize over standard low-rank matrix completion models,[42,43] which might explain why the GSEM outperforms previous approaches that have been proposed to predict drug side effects based on low-rank matrix decomposition.[16–18] Self-representations naturally allow the integration of graph-based information about drugs or side effects. Our model is also related to non-negative MF (NMF).[27,44] They differ, however, in two main aspects. First, while NMF learns two low-rank matrices to represent the input data, the GSEM learns a single null-diagonal matrix that allows for a high-rank matrix.[38] Second, while the NMF objective function is non-convex, we proved that our objective function is convex and that our algorithm converges to a globally optimal solution.

Our framework could be easily applied to proprietary datasets of drug side effects by following our procedure illustrated in Figure 2. The GSEM is fast to run, and its prediction performance is robust to the specific choice of hyperparameters (see our analysis in Figure S5). Applying our model for a compound undergoing clinical trials is as easy as adding the new compound information in a new row in X. We started investigating the potential of the GSEM for drug repositioning,[45] and we envision applying our algorithm to other open problems in biology, chemistry, and medicine, such as drug target prediction[46] or antiviral drug effect prediction.[47] To assist scientists working in clinical drug development in their difficult task, we provide the code to run our algorithm (https://github.com/paccanarolab/GSEM), the predictions for the 505 drugs used in our study (supplementary dataset 4 in Galeano and Paccanaro[48]), and the learned matrices that can help to interpret the predictions (supplementary datasets 5 and 6 in Galeano and Paccanaro[48]).

Whenever machine learning models support high-stakes decisions, it is desirable to have inherently interpretable models.[49] We have shown that the learned matrices in our model capture biological and pharmacological relationships between drugs and physiological relationships between side effect phenotypes. However, the medical, biological, or pharmacological interpretation of the relationships requires expert biological and medical knowledge. In the supplemental information, we also discussed the differences between the interpretability capabilities of the GSEM and our latent factor model for predicting the frequencies of drug side effects[28] (see Methods S3).

Limitations of the study

We run our method only for drugs with at least five side effects identified in clinical trials. A limitation of expanding our analysis is the lack of standardized datasets that classify side effects depending on the phase of the clinical trial in which it was identified.

STAR★METHODS

Detailed methods are provided in the online version of this paper and include the following:

- KEY RESOURCES TABLE
- RESOURCE AVAILABILITY
  - Lead contact
  - Materials availability
  - Data and code availability
- METHOD DETAILS
  - Datasets
  - Side effect ratio of reporting frequency (RRF)
  - Similarities in side information graphs
  - Model selection and evaluation for multiple drugs
  - Performance evaluation for single drugs
  - Multiplicative learning algorithm
- QUANTIFICATION AND STATISTICAL ANALYSIS

SUPPLEMENTAL INFORMATION

Supplemental information can be found online at https://doi.org/10.1016/j.crmeth.2022.100358.

ACKNOWLEDGMENTS

We thank Mateo Torres, Suzana de Siqueira Santos, Ruben Jimenez, Santiago Noto, and Philip Ovington for useful discussions. D.G. was supported by the 2022 KBR SMS SDI Lymphoma grant and the US Air Force grant contract no. FA8075-16-D-0010, task order FA8075-18-F-1690 Explainable Artificial Intelligent Applications within Integrated Dynamic Visualization Environment, and the Facultad de Ingenieria - UNA. A.P. was supported by Biotechnology and Biological Sciences Research Council (https://bbsrc.ukri.org/) grant numbers BB/K004131/1, BB/F00964X/1,and BB/M025047/1; Medical Research Council (https://mrc.ukri.org) grant number MR/T001070/1; Consejo Nacional de Ciencia y Tecnología Paraguay (https://www.conacyt.gov.py/) grants numbers 14-INV-088, PINV15–315, and PINV20-337; National Science Foundation Advances in Bio Informatics (https://www.nsf.gov/) grant number 1660648; Fundação de Amparo à Pesquisa do Estado do Rio de Janeiro grant number E-26/201.079/2021 (260380); and Fundação Getulio Vargas.

AUTHOR CONTRIBUTIONS

Conceptualization, D.G.; methodology, D.G. and A.P.; investigation, D.G.; formal analysis, D.G.; writing – original draft, D.G.; writing – review & editing, D.G. and A.P.; software, D.G.; supervision, A.P.

DECLARATION OF INTERESTS

The authors declare no competing interests.

Received: May 12, 2022
Revised: September 8, 2022
Accepted: November 11, 2022
Published: December 7, 2022

REFERENCES

1. GBD 2016 Causes of Death Collaborators; Abajobir, A.A., Abbafati, C., Abbas, K.M., Abd-Allah, F., Abera, S.F., Aboyans, V., Adetokunboh, O., Afshin, A., Agrawal, A., et al. (2017). Global, regional, and national age-sex specific mortality for 264 causes of death, 1980–2016: a systematic analysis for the global burden of disease study 2016. Lancet *390*, 1151–1210.
2. Sunshine, J.E., Meo, N., Kassebaum, N.J., Collison, M.L., Mokdad, A.H., and Naghavi, M. (2019). Association of adverse effects of medical treatment with mortality in the United States: a secondary analysis of the global burden of diseases, injuries, and risk factors study. JAMA Netw. Open *2*, e187041.

3. Martin, L., Hutchens, M., Hawkins, C., and Radnov, A. (2017). How much do clinical trials cost? Nat. Rev. Drug Discov. *16*, 381–382.
4. Onakpoya, I.J., Heneghan, C.J., and Aronson, J.K. (2016). Post-marketing withdrawal of 462 medicinal products because of adverse drug reactions: a systematic review of the world literature. BMC Med. *14*, 10.
5. Ho, T.-B., Le, L., Thai, D.T., and Taewijit, S. (2016). Data-driven approach to detect and predict adverse drug reactions. Curr. Pharm. Des. *22*, 3498–3526.
6. Boland, M.R., Jacunski, A., Lorberbaum, T., Romano, J.D., Moskovitch, R., and Tatonetti, N.P. (2016). Systems biology approaches for identifying adverse drug reactions and elucidating their underlying biological mechanisms. Wiley Interdiscip. Rev. Syst. Biol. Med. *8*, 104–122.
7. Yamanishi, Y., Pauwels, E., and Kotera, M. (2012). Drug side-effect prediction based on the integration of chemical and biological spaces. J. Chem. Inf. Model. *52*, 3284–3292.
8. Fliri, A.F., Loging, W.T., Thadeio, P.F., and Volkmann, R.A. (2005). Analysis of drug-induced effect patterns to link structure and side effects of medicines. Nat. Chem. Biol. *1*, 389–397.
9. Atias, N., and Sharan, R. (2011). An algorithmic framework for predicting side effects of drugs. J. Comput. Biol. *18*, 207–218.
10. Lounkine, E., Keiser, M.J., Whitebread, S., Mikhailov, D., Hamon, J., Jenkins, J.L., Lavan, P., Weber, E., Doak, A.K., Côté, S., et al. (2012). Large-scale prediction and testing of drug activity on side-effect targets. Nature *486*, 361–367.
11. Poleksic, A., and Xie, L. (2018). Predicting serious rare adverse reactions of novel chemicals. Bioinformatics *34*, 2835–2842.
12. LaBute, M.X., Zhang, X., Lenderman, J., Bennion, B.J., Wong, S.E., and Lightstone, F.C. (2014). Adverse drug reaction prediction using scores produced by large-scale drug-protein target docking on high-performance computing machines. PLoS One *9*. e106298.
13. Scheiber, J., Chen, B., Milik, M., Sukuru, S.C.K., Bender, A., Mikhailov, D., Whitebread, S., Hamon, J., Azzaoui, K., Urban, L., et al. (2009). Gaining insight into off-target mediated effects of drug candidates with a comprehensive systems chemical biology analysis. J. Chem. Inf. Model. *49*, 308–317.
14. Zhou, H., Gao, M., and Skolnick, J. (2015). Comprehensive prediction of drug-protein interactions and side effects for the human proteome. Sci. Rep. *5*, 11090.
15. Cami, A., Arnold, A., Manzi, S., and Reis, B. (2011). Predicting adverse drug events using pharmacological network models. Sci. Transl. Med. *3*. 114ra127.
16. Galeano, D., and Paccanaro, A. (2018). A recommender system approach for predicting drug side effects. In 2018 International Joint Conference on Neural Networks (IJCNN) (IEEE), pp. 1–8.
17. Li, R., Dong, Y., Kuang, Q., Wu, Y., Li, Y., Zhu, M., and Li, M. (2015). Inductive matrix completion for predicting adverse drug reactions (adrs) integrating drug–target interactions. Chemometr. Intell. Lab. Syst. *144*, 71–79.
18. Zhang, W., Liu, X., Chen, Y., Wu, W., Wang, W., and Li, X. (2018). Feature-derived graph regularized matrix factorization for predicting drug side effects. Neurocomputing *287*, 154–162.
19. Bean, D.M., Wu, H., Iqbal, E., Dzahini, O., Ibrahim, Z.M., Broadbent, M., Stewart, R., and Dobson, R.J.B. (2018). Knowledge graph prediction of unknown adverse drug reactions and validation in electronic health records. Sci. Rep. *8*, 4284.
20. Knepper, T.C., and McLeod, H.L. (2018). When will clinical trials finally reflect diversity? Nature *557*, 157–159.
21. Kuhn, M., Letunic, I., Jensen, L.J., and Bork, P. (2016). The sider database of drugs and side effects. Nucleic Acids Res. *44*, D1075–D1079.
22. Ng, A.Y. (2004). Feature selection, l 1 vs. l 2 regularization, and rotational invariance. In Proceedings of the Twenty-First International Conference on Machine Learning (ACM), p. 78.
23. Zou, H., and Hastie, T. (2005). Regularization and variable selection via the elastic net. J. Royal Statistical Soc. B *67*, 301–320.
24. Ma, H., Zhou, D., Liu, C., Lyu, M.R., and King, I. (2011). Recommender systems with social regularization. In Proceedings of the fourth ACM international conference on Web search and data mining (ACM), pp. 287–296.
25. Kalofolias, V., Bresson, X., Bronstein, M., and Vandergheynst, P. (2014). Matrix completion on graphs. Preprint at arXiv. https://doi.org/10.48550/arXiv.1408.1717.
26. Monti, F., Bronstein, M., and Bresson, X. (2017). Geometric matrix completion with recurrent multi-graph neural networks. In Advances in Neural Information Processing Systems, pp. 3697–3707.
27. Lee, D.D., and Seung, H.S. (1999). Learning the parts of objects by non-negative matrix factorization. Nature *401*, 788–791.
28. Galeano, D., Li, S., Gerstein, M., and Paccanaro, A. (2020). Predicting the frequencies of drug side effects. Nat. Commun. *11*, 4575.
29. Tatonetti, N.P., Ye, P.P., Daneshjou, R., and Altman, R.B. (2012). Data-driven prediction of drug effects and interactions. Sci. Transl. Med. *4*. 125ra31.
30. Wishart, D.S., Feunang, Y.D., Guo, A.C., Lo, E.J., Marcu, A., Grant, J.R., Sajed, T., Johnson, D., Li, C., Sayeeda, Z., et al. (2018). Drugbank 5.0: a major update to the drugbank database for 2018. Nucleic Acids Res. *46*, D1074–D1082.
31. Corsello, S.M., Bittker, J.A., Liu, Z., Gould, J., McCarren, P., Hirschman, J.E., Johnston, S.E., Vrcic, A., Wong, B., Khan, M., et al. (2017). The drug repurposing hub: a next-generation drug library and information resource. Nat. Med. *23*, 405–408.
32. MDL Information Systems/Symyx (1984). MACCS-II (MDL Information Systems/Symyx).
33. Landrum, G. (2013). Rdkit: A Software Suite for Cheminformatics, Computational Chemistry, and Predictive Modeling (Sourceforge).
34. Bishop, C.M. (2006). Pattern Recognition and Machine Learning (Springer).
35. Hinton, G. (2018). Deep learning—a technology with the potential to transform health care. JAMA *320*, 1101–1102.
36. Cheng, F., Kovács, I.A., and Barabási, A.L. (2019). Network-based prediction of drug combinations. Nat. Commun. *10*, 1197.
37. Behar, S., Reicher-Reiss, H., Abinader, E., Agmon, J., Friedman, Y., Barzilai, J., Kaplinsky, E., Kauli, N., Kishon, Y., Palant, A., et al. (1992). The prognostic significance of angina pectoris preceding the occurrence of a first acute myocardial infarction in 4166 consecutive hospitalized patients. Am. Heart J. *123*, 1481–1486.
38. Elhamifar, E. (2016). High-rank matrix completion and clustering under self-expressive models. In Advances in Neural Information Processing Systems, pp. 73–81.
39. Ning, X., and Karypis, G. (2011). Slim: sparse linear methods for top-n recommender systems. In Data Mining (ICDM), 2011 IEEE 11th International Conference (IEEE), pp. 497–506.
40. Bronstein, M.M., Bruna, J., LeCun, Y., Szlam, A., and Vandergheynst, P. (2017). Geometric deep learning: going beyond euclidean data. IEEE Signal Process. Mag. *34*, 18–42.
41. Hamilton, W.L., Ying, R., and Leskovec, J. (2017). Representation learning on graphs: methods and applications. IEEE Data Eng. Bull. *40*, 52–74.
42. Fan, J., and Chow, T.W. (2017). Matrix completion by least-square, low-rank, and sparse self-representations. Pattern Recogn. *71*, 290–305.
43. Wang, Y., and Elhamifar, E. (2018). High rank matrix completion with side information. In Thirty-Second AAAI Conference on Artificial Intelligence.
44. Lee, D.D., and Seung, H.S. (2001). Algorithms for non-negative matrix factorization. In Advances in neural information processing systems, pp. 556–562.
45. Frasca, F., Galeano, D., Gonzalez, G., Laponogov, I., Veselkov, K., Paccanaro, A., and Bronstein, M.M. (2019). Learning interpretable disease

self-representations for drug repositioning. Preprint at arXiv. https://doi.org/10.48550/arXiv.1909.06609.

46. Simm, J., Arany, A., Zakeri, P., Haber, T., Wegner, J.K., Chupakhin, V., Ceulemans, H., and Moreau, Y. (2017). Macau: scalable bayesian factorization with high-dimensional side information using mcmc. In 2017 IEEE 27th International Workshop on Machine Learning for Signal Processing (MLSP) (IEEE), pp. 1–6.

47. Santos, S.d.S., Torres, M., Galeano, D., Sánchez, M.D.M., Cernuzzi, L., and Paccanaro, A. (2022). Machine learning and network medicine approaches for drug repositioning for covid-19. Patterns *3*, 100396.

48. Galeano, D., and Paccanaro, A.. (2022). Machine Learning Prediction of Side effects for Drugs in Clinical Trials - Galeano and Paccanaro. Mendeley. https://doi.org/10.17632/3z7c4r52n3.1.

49. Rudin, C. (2019). Stop explaining black box machine learning models for high stakes decisions and use interpretable models instead. Nat. Mach. Intell. *1*, 206–215.

50. Campillos, M., Kuhn, M., Gavin, A.-C., Jensen, L.J., and Bork, P. (2008). Drug target identification using side-effect similarity. Science *321*, 263–266.

STAR★METHODS

KEY RESOURCES TABLE

REAGENT or RESOURCE	SOURCE	IDENTIFIER
Deposited data		
SIDER 4.1	Kuhn et al.[21]	http://sideeffects.embl.de/
OFFSIDES	Tatonetti et al.[29]	https://tatonettilab.org/offsides/
DrugBank	Wishart et al.[30]	v 5.1. https://go.drugbank.com/
Drug Repurposing Hub	Corsello et al.[31]	version 3/24/2020 https://clue.io/repurposing
Anatomical, Therapeutic, and Chemical classification (ATC) code	https://www.whocc.no/atc_ddd_index/	commercial release 2018
Supplementary dataset 1. Drug side effect dataset.	Galeano et al.[28]	Mendeley Data[48]
Supplementary dataset 2. Drug similarity networks.	N/A	Mendeley Data[48]
Supplementary dataset 3. Side effect similarity networks.	N/A	Mendeley Data[48]
Supplementary dataset 4. Predicted GSEM scores	N/A	Mendeley Data[48]
Supplementary dataset 5. Learned H	N/A	Mendeley Data[48]
Supplementary dataset 6. Learned W	N/A	Mendeley Data[48]
Software and algorithms		
GSEM algorithm	https://github.com/paccanarolab/GSEM	Zenodo (https://doi.org/10.5281/zenodo.7291925)
MATLAB R2022a	https://www.mathworks.com/products/matlab.html	Copyright 1993-2020 The MathWorks, Inc.

RESOURCE AVAILABILITY

Lead contact

Requests for further information should be directed to the lead contact, Diego Galeano (dgaleano@ing.una.py).

Materials availability

This study did not generate new materials.

Data and code availability

- This paper analyzes existing, publicly available data. All the datasets used and generated in our study were deposited in Mendeley Data (https://doi.org/10.17632/3z7c4r52n3.1) and they are publicly available as of the date of publication. Datasets include supplementary dataset 1-6 from Galeano and Paccanaro.[50]
- All original code has been deposited at GitHub (https://github.com/paccanarolab/GSEM) and is publicly available as of the date of publication.
- Any additional information required to reanalyze the data reported in this paper is available from the lead contact upon request.

METHOD DETAILS

Datasets

Clinical side effects

We used the dataset collected in our previous study of the frequencies of drug side effects.[28] Clinical side effects correspond to those drug side effect associations with an associated frequency from randomized controlled studies in the Side effect Resource (SIDER) database version 4.1[21]. 27,610 associations were found between the 505 drugs and 904 unique side effect terms. Each side effect term was mapped to a Medical Dictionary for Regularity Activities (MedDRA) Preferred-Term. A detailed explanation of the data processing can be found in the Supplementary Note 1 in Galeano et al.[28]

Postmarketing side effects

Two test sets of postmarketing side effects were collected. The first set was obtained from the SIDER 4.1 database,[21] from which we retrieved 6,818 postmarket associations (labels 'postmarketing' in SIDER) – it corresponds to side effects reported in the postmarketing section of drug's leaflets. The second set was obtained from the OFFSIDES database,[29] from which we retrieved 25,797 "significant" associations - it corresponds to statistically significant postmarketing side effects reported in the Adverse Event Reporting System (AERS).

Drug-target interactions

We retrieved the known drug-target interactions from DrugBank release 5.1[30]. We mapped the drugs from SIDER to DrugBank using the PubChem IDs and the mapping provided in DrugBank. We retrieved molecular targets (section 'targets' of DrugBank) for the 505 drugs in our dataset. In total, 1,983 associations were found between the 505 drugs and 755 unique protein targets.

Chemical fingerprints

We retrieved the known drug SMILES notations from DrugBank release 5.1[30]. For the 505 drugs in our dataset, we could obtain a binary MACCS fingerprint using the open source RDKit python library.[33] MACCS are 167 bit structural key descriptors in which each bit is associated with an SMARTS pattern.[32]

Drug indications

We retrieved drug indications from the Drug Repositioning Hub database[31] (accessed on 05/02/2020). Drug indications in the Drug Repositioning Hub were manually annotated. In total, 1,021 associations were found between the 505 drugs and 354 unique indications.

ATC information

We retrieved Anatomical, Therapeutic and Chemical (ATC) codes for each of the 505 drugs from the WHO proprietary dataset release 2018.

The datasets and similarity values used to implement GSEM are provided in Supplementary Dataset 1, 2 and 3.

Side effect ratio of reporting frequency (RRF)

The side effect ratio of reporting frequency is a normalized count of the number of drugs associated with a given side effect. For a given side effect j, the $RRF(j)$ is defined as follow:

$$RRF(j) = \frac{\sum_i^n x_{ij}}{Z} \quad \text{(Equation 6)}$$

where x_{ij} in the entry (i,j) of the matrix X, n is the total number of drugs, and $Z = \max\{\sum_i^n x_{i1}, \sum_i^n x_{i2}, \ldots, \sum_i^n x_{im}\}$ is the maximum number of associations for the side effects. When using only drug side effect associations from clinical trials, $Z = 375$.

Similarities in side information graphs

To build the graphs for drugs, we computed similarities from the side information features. Given a set of feature elements U associated with drug u (e.g. chemical fingerprints) and a set of feature elements V associated with drug v, the Jaccard similarity between u and v is given by:

$$\mathcal{J}(u,v) = \frac{|V \cap U|}{|V \cup U|} \quad \text{(Equation 7)}$$

where $|.|$ denotes the cardinality of the sets. The Jaccard similarity is bounded $0 \leq \mathcal{J}(u,v) \leq 1$.

The Jaccard similarity was used for the chemical, indication and target drug features. Three weighted and symmetric adjacency matrices $A_{chem}, A_{Ind}, A_{DT}$ were then obtained for each side information type. The Jaccard similarity of the chemical fingerprints is also known as the 2D Tanimoto Chemical similarity.

For the ATC side information, we followed Cami et al.[15] and calculated taxonomy similarities between drugs based on the shortest path between their set of ATC codes in the ATC hierarchy. ATC has four different levels, and each drug was annotated by its corresponding ATC codes in the lower level of the hierarchy. Given two drugs u and v, the ATC taxonomy similarity between the drugs was then calculated as follow:

$$TAX_{ATC}(u,v) = 1 - \frac{SP(ATC_u, ATC_v)}{\max_{(i,j) \in \Omega} SP(ATC_i, ATC_j)} \quad \text{(Equation 8)}$$

where ATC_u and ATC_u correspond to the set of ATC codes of drug u and v, respectively; $SP(ATC_u, ATC_v)$ is a function that calculates the shortest path between the set of ATC annotations; and Ω is the set of drugs. In the ATC hierarchy, the smallest value of the shortest path between drugs is 2 and the largest is 8. The ATC taxonomy similarity between two drugs is a number between 0 and 1. We also obtained an adjacency matrix between drugs (A_{ATC}) based on the ATC taxonomy similarity.

In total, four drug graphs were used in our model in Equation 2. The adjacency matrices for each of those graphs correspond to $A_{chem}, A_{Ind}, A_{DT}$ and A_{ATC}. For side effects, we used one side information only in Equation 3. We computed the MedDRA taxonomy similarity using the MedDRA hierarchy following the same procedure used for calculating the ATC taxonomy similarity.

Model selection and evaluation for multiple drugs

To evaluate each model for multiple drugs, we built a held-out test set by randomly sampling 10% of the known associations in X containing clinical trials side effects. The held-out test set contained 2,761 associations (positive class). To obtain the zeros for the test set (negative class), we randomly sampled twice the number of positives from the zero entries of X that were not in the test set. To set each of the model parameters, we randomly sampled 10% of the remaining entries in X, and placed them on a validation set. The negative class for the validation set was also build by the same negative sampling procedure used for the held-out test set. The validation set contained 2,484 associations and the training set contained 22,365 associations. We used the validation set for model selection. Model parameters were selected according to the Area Under the Receiver Operating Curve (AUROC) in the validation set. The details of the implementation of each model and the grid search for the model parameters is explained in Methods S1.

To assess the performance of the models in the held-out test set, we used the best set of parameters for each model and re-trained the models using all the combined training and validation sets. Then, the model was used to assess the performance in the held-out test set. To assess the performance of the model in the postmarketing test sets, we trained the model with the best set of parameters obtained from the validation set and by considering all the available data from clinical trials, that is, a total of 27,610 associations. In our evaluations for multiple drugs, we also reported the Area Under the Precision-Recall Curve (AUPR).

Performance evaluation for single drugs

When evaluating the performance of our method on single drugs, we trained the model using the following parameters $a = 60$, $b = 0, \mu_{chem} = 0.1, \mu_{Ind} = 0.5, \mu_{DT} = 0.01, \mu_{ATC} = 5, \gamma = 10^4, c = 40, d = 0.5$, $\alpha_{MedDRA} = 0.5$. The procedure for each case presented in the manuscript is as follow:

Evaluation by groups of drugs

We trained our method using only clinical trials side effects. The performance of the model was then assessed for each drug on whether the model was able to predict the postmarketing side effects from of all the possible side effects – these correspond to the entries in a row of X that had values of zeros in training. We performed this evaluation for drugs with at least ten associations in the testing sets. We used the area under the receiving operating curve (AUROC) to measure the performance of the model. The performance was then reported by grouping drugs according to their main Anatomical, Therapeutic and Chemical (ATC) categories.

Evaluation by groups of side effects

We followed the same procedure described for groups of drugs. The difference is that for side effects, we assessed the performance for each side effect, by predicting postmarketing associations for a given column of X. The performance was then reported by grouping side effects based on their main MedDRA category of disorders.

Evaluation when including postmarketing associations in training

For each drug, we used its clinical trials side effects for training and used its SIDER and OFFSIDES postmarketing side effects for testing. For the remaining drugs in X, we also included its SIDER and OFFSIDES postmarketing associations. To prevent biases in the evaluation due to the presence of drug analogs, we removed the drugs in X that were above a Tanimoto chemical similarity threshold of 0.6 – this threshold had been used before to separate chemically similar from dissimilar drugs.[50]

Multiplicative learning algorithm

To minimize Equations 2 and 3 subject to the non-negative constraints $W, H \geq 0$, we developed efficient multiplicative algorithms inspired by the diagonally re-scaled principle of non-negative matrix factorization.[27,44] The algorithm consists in iteratively applying the following multiplicative update rules:

$$w_{ij} \leftarrow w_{ij} \frac{\left(X^T X + \sum_k \mu_k W A_k\right)_{ij}}{\left(X^T X W + \sum_k \mu_k W D_k + aW + b + \gamma I\right)_{ij}} \qquad \text{(Equation 9)}$$

$$h_{ij} \leftarrow h_{ij} \frac{\left(X X^T + \alpha A_{MedDRA} H\right)_{ij}}{\left(X X^T H + \alpha D_{MedDRA} H + cH + d + \gamma I\right)_{ij}} \qquad \text{(Equation 10)}$$

where W and H are initialized as random dense matrices uniformly distributed in the range $[0, 0.01]$. The stopping criteria of our algorithm was based on the maximum tolerance of the relative change in the elements of W and H. The default value was $\text{TolX} < 10^{-2}$, that occurred typically in about 50 iterations.

We proved that the iterative application of Equations 9 and 10 converges to a global optimal solution point by showing that the multiplicative learning rule satisfies the Karush-Khun-Tucker (KKT) conditions of convergence and that the objective functions are convex (Proofs in Methods S2).

Self-representation similarity

Given the drug self-representation matrix H, we defined the similarity between drugs as follow:

$$S_H = \left(H + H^T\right)/2 \qquad \text{(Equation 11)}$$

The similarity between side effects was defined similarly:

$$S_W = (W + W^T)/2 \quad \text{(Equation 12)}$$

Interpretability procedure

Following Cheng et al.,[36] we analyzed whether the drug self-representation similarities, as captured by S_H, capture the known chemical, biological and pharmacological relationships between drugs. For chemical relationships we used the 2D Tanimoto chemical similarity between drugs, for biological we used drug targets similarities, and for pharmacological relationship, we used the ATC Taxonomy and indications similarities. We also analyzed whether the side effect self-representation similarities, as captured by S_W, reflects the physiological relationship between the side effect phenotypes. For this analysis, we used the MedDRA taxonomy similarity.

To analyze the self-representations, we trained our model without side information graphs, i.e. with the parameters $a = 70, b = 0, \mu_{chem} = 0, \mu_{Ind} = 0, \mu_{DT} = 0, \mu_{ATC} = 0, \gamma = 10^4, c = 30, d = 0.5, \alpha_{MedDRA} = 0$. We trained the model using all the available data (clinical trials and postmarketing side effects), that is, using 59,497 associations. We then binned the drug and side effect self-representation similarity matrices, S_W and S_H, and checked the values of the side information similarities corresponding to each specific bin. The bins used were $0 - 0.05, 0.05 - 0.1, 0.1 - 0.2$ and > 0.2.

QUANTIFICATION AND STATISTICAL ANALYSIS

One-tailed Wilcoxon Sum Rank Test Significance was used in the reported *P*-values. To analyze the significance of the RRF values for a given drug or side effect category, we adjusted the p values using the Benjamini-Hochberg method to keep the overall significance level below 0.05.

Cell Reports Methods, Volume *2*

Supplemental information

Machine learning prediction of side effects for drugs in clinical trials

Diego Galeano and Alberto Paccanaro

Supplemental information

Machine Learning Prediction of Side effects for Drugs in Clinical Trials

Diego Galeano, Alberto Paccanaro

Methods S1. Methods implementation and optimization. Related to STAR Methods.

Predictive Pharmacosafety Networks (PPNs)

PPNs[1] represents the drug side effect associations as a bipartite graph and topological features are extracted from the network for the prediction. PPNs model the binary response variable $X_{uj}, u \in \{1, ..., n\}, j \in \{1, ..., m\}$, for n drugs and m side effects, denoting the presence or absence of drug side effect associations. Using Logistic Regression (LR), PPNs modelled this response as a Bernoulli random variable with the following expectation:

$$E[X_{uj}] = \frac{1}{1 + \exp\left(-\sum_s \beta_s Z_s(u, j)\right)} \tag{1}$$

Here, β_s denotes the model parameter and Z_s the model covariate. PPNs extracts network covariates as well as other covariates from intrinsic, biological and taxonomic side information.

Network Covariates

In the following, let u represent a drug node, and j a side effect node. Then, the degree covariates are defined as follow:

$$\begin{aligned} Z_1(u, j) &= \deg(u) \times \deg(j) \\ Z_2(u, j) &= |\deg(u) - \deg(j)| \\ Z_3(u, j) &= \deg(u) + \deg(j) \\ Z_4(u, j) &= \frac{\deg(u)}{\deg(j)} \end{aligned} \tag{2}$$

Here, $\deg(u)$ denotes the degree of node u. The degree product covariate, $Z_1(u, j)$, aims to capture preferential attachment among high-degree drugs and side effects. The degree difference covariate, $Z_2(u, j)$, aims to capture assortativity, i.e. whether high-degree drugs connects to high-degree side effects or to small-degree side effects.

Further, distance covariates accounts for the set of neighbours of drug u, that we denoted as $\mathcal{N}(u)$, and the set of neighbours of side effect j, denoted as $\mathcal{N}(j)$. Let also $J(j, k)$ denotes the Jaccard similarity between the neighbours sets $\mathcal{N}(j)$ and $\mathcal{N}(k)$, which is defined as follow:

$$J(j, k) = \frac{|\mathcal{N}(j) \cap \mathcal{N}(k)|}{|\mathcal{N}(j) \cup \mathcal{N}(k)|} \tag{3}$$

The Jaccard-based covariates quantify structural similarity between drug pairs and side effect pairs:

$$\begin{aligned} Z_5(u, j) &= \max_{k \in \mathcal{N}(u) - \{j\}} \{J(j, k)\} \\ Z_6(u, j) &= \max_{k \in \mathcal{N}(j) - \{u\}} \{J(u, k)\} \end{aligned} \tag{4}$$

Finally, Jaccard-based predictors based on Kullback-Leibler divergence ($\mathcal{K}_\mathcal{L}$) between the overall distribution of similarities between a drug ($\bar{D}_{se}$) and the drugs in its local neighbourhood ($D_{se}(u, j)$) or between side effects ($\bar{D}_{se}$) and side effects in its neighbourhood ($D_{se}(u, j)$) are defined as follow:

$$\begin{aligned} Z_7(u, j) &= \mathcal{K}_\mathcal{L}(D_{se}(u, j), \bar{D}_{se}) \\ Z_8(u, j) &= \mathcal{K}_\mathcal{L}(D_{drug}(u, j), \bar{D}_{drug}) \end{aligned} \tag{5}$$

Intrinsic, biological and taxonomic covariates

Given additional information about drugs (or side effects), these can also be included in the prediction. Intrinsic covariates are those that are defined based on the intrinsic properties of drugs, i.e. chemical structure. For instance, let $d_{chem}(u,k)$ represent a chemical distance between two given drugs u and k, then, the intrinsic covariate is defined as:

$$\begin{aligned} Z_9(u,j) &= \min_{k \in \mathcal{N}(j)-\{i\}} \{d_{chem}(u,k)\} \\ Z_{10}(u,j) &= \min_{k \in \mathcal{N}(j)-\{i\}} \{d_{DT}(u,k)\} \\ Z_{11}(u,j) &= \min_{k \in \mathcal{N}(j)-\{i\}} \{d_{ATC}(u,k)\} \\ Z_{12}(u,j) &= \min_{k \in \mathcal{N}(j)-\{i\}} \{d_{MedDRA}(u,k)\} \end{aligned} \tag{6}$$

We also use the other covariates presented in[1]. These include (i) intrinsic, defined by our 2D Tanimoto chemical similarity; (ii) biological, defined by drug target similarity and (iii) taxonomy, defined by our ATC and MedDRA taxonomy similarities. For PPNs, all the similarities were converted into distances by subtracting one from each similarity matrix.

Optimisation We use the Matlab built-in function `mnrfit` (link function `logit`) to learn the coefficients estimates (β_s) of the multinomial logistic regression of the nominal responses in X on the predictors in Z.

Hyperparameters tunning PPNs does not require hyperparameters tunning.

Low-rank matrix factorization (MF)

The MF model[2] is based on the assumption that the binary matrix $X \in R^{n \times m}$ for n drugs and m side effects of rank $k \ll min\{m,n\}$, can be expressed as the product of two matrices of rank-k, as follow:

$$X \approx PQ \tag{7}$$

here $P \in R^{n \times k}$ and $Q \in R^{k \times m}$. This model amounts to assign a low-dimensional feature vector to each drug and a low-dimensional feature vector to each side effect such that the dot-product between the features vectors is related to the probability that a drug is associated to a side effect. The rank of X is k — the number of features of each drug and side effect. The matrices P and Q are the solution of the following optimisation problem:

$$\min_{P,Q} \mathcal{L}(P,Q) = \underbrace{\frac{1}{2}\|X - PQ\|_F^2}_{\text{low-rank model}} + \underbrace{\frac{\lambda}{2}(\|P\|_F^2 + \|Q\|_F^2)}_{\text{regularisation}} \tag{8}$$

where $\|.\|_F$ is the Frobenius, and the term $\lambda(\|P\|_F^2 + \|Q\|_F^2)$ is a $\mathcal{L}_2$ regularisation term with penalty $\lambda > 0$, which is added to the objective function in order to prevent overfitting.

The problem is non-convex in both P and Q. Gradient descent-based optimisation methods are required to approximate the solution. Thus, the derivatives of (8) are:

$$\frac{\partial \mathcal{L}(P,Q)}{\partial P} = -(X - PQ)Q^T + \lambda P \tag{9}$$

$$\frac{\partial \mathcal{L}(P,Q)}{\partial Q} = -P^T(X - PQ) + \lambda Q \tag{10}$$

Optimisation We used the code provided by the authors[2]. The optimisation was done using conjugate gradient descent (CGD) and approximated line searches based on polynomial interpolation with Wolfe-Powel conditions to find the local minima in (8).

Hyperparameters tunning Following the procedure presented in[2], we initialize P and Q as normally distributed random variables with small variance $\sigma^2 = 0.01$. We tuned both model parameters: the number of latent factors k and the regularisation penalty λ in the grid: $\lambda \in \{1, 5, 10, 15, 20\}$ and $k \in \{1, 5, 10, 20, 30, 40, 50, 60, 70, 80, 90, 100\}$.

Inductive Matrix Completion (IMC)

IMC[3] is a low-rank model that integrates drug and side effect attributes. For the binary matrix $X \in R^{n \times m}$ for n drugs and m side effects, let $K_d \in R^{n \times n}$ represent the kernel similarity matrix for drugs and, similarly, let $K_a \in R^{m \times m}$ represent the kernel similarity matrix for side effects. When only drug or side effect attributes are available, the kernel matrices are built using similarity measures. IMC aims to approximate X by:

$$X \simeq K_d W H K_a \tag{11}$$

By minimising the following loss function:

$$\min_{W,H} \mathcal{L}(W,H) = \frac{1}{2}\|\Omega \circ (X - K_d W H K_a)\|_F^2 + \frac{\lambda}{2}(\|W\|_F^2 + \|H\|_F^2) \tag{12}$$

Here, Ω represents a projection over the observed entries in X, $\circ$ is the element-wise product between matrices, $W \in R^{n \times k}$ is the drug latent representation matrix and $H \in R^{k \times m}$ is the side effect latent representation matrix. The second term in Eq. 12 is a $\mathcal{L}_2$ regularisation term applied to the latent matrices W and H, with a positive penalty $\lambda > 0$.

Gradient descent-based methods are required to approximate the solution. Thus, the derivatives of (12) are:

$$\frac{\partial \mathcal{L}(W,H)}{\partial W} = -K_d(X - \Omega \circ (K_d W H K_a))K_a H^T + \lambda W \tag{13}$$

$$\frac{\partial \mathcal{L}(W,H)}{\partial H} = -W^T K_d(X - \Omega \circ (K_d W H K_a))K_a + \lambda H \tag{14}$$

IMC uses chemical and drug target similarities for K_d and MedDRA taxonomic similarities for K_a. Following the procedure in[3], we used $K_d = \frac{1}{2}(A_{chem} + A_{DT})$ and $K_a = A_{MedDRA}$ as a combination model.

Optimisation We used conjugate gradient descent (CGD) and approximated line searches based on polynomial interpolation with Wolfe-Powel conditions to find the local minima in (12).

Hyperparameters tunning We initialize P and Q as normally distributed random variables with small variance $\sigma^2 = 0.01$. We tuned both model parameters: the number of latent factors k and the regularization penalty λ in the grid: $k \in \{1, 5, 10, 20, 30, 40, 50, 60, 70, 80, 90, 100\}$ and $\lambda \in \{1, 5, 10, 15, 20, 30, 40, 50\}$.

Feature-derived graph regularized matrix factorization (FGRMF)

The FGRMF[4] integrates several low-rank models, one per side information graph, by using a logistic regression model. Each low-rank model is optimised independently and then combined. For the binary matrix $X \in R^{n \times m}$ for n drugs and m side effects, FGRMF first minimises the following loss:

$$\min_{W,H} \mathcal{L}(W,H) = \underbrace{\frac{1}{2}\|X - WH\|_F^2}_{\text{low-rank model}} + \underbrace{\frac{\lambda}{2}(\|W\|_F^2 + \|H\|_F^2)}_{\text{regularisation}} + \underbrace{\frac{\alpha}{2}\|W\|_{\mathcal{D},\mathcal{G}}^2}_{\text{smoothness}} \tag{15}$$

where $\|.\|_F$ denotes the Frobenius norm, and $\|C\|_{\mathcal{D},\mathcal{G}}$ the Dirichlet norm on the graph $\mathcal{G} = (\{1,\dots,n\},\mathcal{E},G)$, i.e. the weighted undirected graph with edge weights $g_{ij} > 0$ if $(i,j) \in \mathcal{E}$ and zero otherwise, representing the similarities between drugs.

The second step of FGRMF is to combined the solution from multiple graphs $\{\mathcal{G}_1, \mathcal{G}_2, ..., \mathcal{G}_p\}$, that gives different approximations to X, $\{\hat{X}_1, \hat{X}_2, ..., \hat{X}_p\}$ as follow:

$$h_\theta = \frac{1}{1 + \exp\left(-(\theta_0 + \sum_{i=1}^p \theta_i \hat{X}_i\right)} \tag{16}$$

where the weights θ_j are learned for each independent model. h_θ is used as a final prediction model.

Optimisation To find the solution in Eq. (15), we implemented the algorithm described by the authors. To optimise Eq. (16), we use the Matlab built-in function `mnrfit` (link function `logit`) to learn the coefficients estimates (θ_j).

Hyperparameters tunning We initialize W and H as normally distributed random variables with small variance $\sigma^2 = 0.01$. We tuned the three model parameters: the number of latent factors k, the regularisation penalty λ and the penalty α in the grid: $k \in \{1,5,10,20,30,40,50,60,70,80,90,100\}$ and $\lambda \in \{1,5,10,20\}$ and $\alpha \in \{0.01,0.5,1,5\}$.

Geometric Self-Expressive Model (GSEM)

Optimisation We followed the recommended guidelines to implement non-negative matrix factorization (NMF)[5]. W and H were initialised with weights from a uniform distribution between $(0,\sqrt{\sigma}]$ for a $\sigma = 0.01$. The maximum number of iterations was set to 500 and `tolX` $= 0.01$. With this `tolX`, convergence occurs in roughly 50 iterations.

Hyperparameters tunning For each GSEM model, we tuned the parameters in the following grid: L2 regularisation parameter $a, c \in \{1,10,20,30,40,50,60,70,80,90,100\}$, L1 regularisation parameter $b, d \in \{0,0.5,1,2,3,4,5\}$, graph regularisation parameter $\mu, \alpha \in \{0.01,0.1,0.5,1,5,10\}$. γ were set to 10^4 for all the experiments (we empirically observed that with this value diag(W)$\simeq 0$).

Computing infrastructure

All the models were run in a small cluster with 32 physical cores and 128GB of RAM. Each processor is a Intel(R) Xeon(R) CPU E5-2683 v4 @ 2.10GHz. We implemented most of the algorithms in Matlab R2019a 64-bit. Training of model hyperparameters was executed in parallel using the Parallel Computing Toolbox version 6.12.

Methods S2. Theoretical guarantees of GSEM. Related to STAR Methods.

Convexity

Here we prove that the GSEMW objective function is *convex*. The same procedure can be used to prove that the GSEMH objective is convex. Let us recall our objective function:

$$\mathcal{L}(W) = \underbrace{\frac{1}{2}\|X - XW\|_F^2}_{\text{self-representation}} + \underbrace{\frac{a}{2}\|W\|_F^2 + b\|W\|_1}_{\text{sparsity}} + \underbrace{\sum_i \frac{\mu_i}{2}\|W\|^2_{\mathcal{D},\mathcal{G}_i}}_{\text{smoothness}} + \underbrace{\gamma \operatorname{Tr}(W)}_{\text{null diagonal}} \tag{17}$$

Theorem 1 (Convexity) *For a real matrix X, the objective function $\mathcal{L}(W)$ is convex in the feasible region $W \geq 0$.*

We need to show that the Hessian of $\mathcal{L}(W)$ is a positive semi-definite (PSD) matrix. The Hessian of W is the second derivative of $\mathcal{L}(W)$, that is:

$$\nabla^2 \mathcal{L}(W) = X^T X + a + \sum_j \mu_j L_j$$

where $L_j = D_j - A_j$ is the graph Laplacian for the jth graph. The Hessian is PSD iif for a non-zero column vector $h \in R^m$ of real numbers the scalar $h^T \nabla^2 \mathcal{L}(W) h \geq 0$, i.e.,

$$\begin{aligned} h^T(X^T X + a + \sum_j \mu_j L_j)h \geq 0 \\ h^T X^T X h + a h^T h + \sum_j h^T \mu_j L_j h \geq 0 \\ \| Xh \|^2 + a \| h \|^2 + \sum_j \mu_j \| y_j \|^2 \geq 0 \quad \forall h, y, a > 0 \end{aligned} \tag{18}$$

In the last step, we use the fact that the Laplacian L_j is PSD and thus can be diagonalised as follow:

$$L_j = U_j^T \Lambda_j U_j = U_j^T \Lambda_j^{\frac{1}{2}} \Lambda_j^{\frac{1}{2}} U = (\Lambda_j^{\frac{1}{2}} U)^T (\Lambda_j^{\frac{1}{2}} U)$$

Letting $y_j = \Lambda_j^{\frac{1}{2}} U$, we see that:

$$h^T \alpha_j L_j h = \alpha_j y_j^T y_j = \alpha_j \| y_j \|^2 \geq 0$$

That is, the GSEMW objective function is convex in W.

Global optima convergence

The Karush-Khun-Tucker (KKT) complementary conditions of convergence are both necessary and sufficient conditions for a global solution point[6], given that the objective function $\mathcal{L}(W)$ is convex. We shall prove that our multiplicative learning algorithm satisfies the KKT conditions.

$$w_{ij} \leftarrow w_{ij} \frac{(X^T X + \sum_k \mu_k W A_k)_{ij}}{(X^T X W + \sum_k \mu_k W D_k + aW + b + \gamma I)_{ij}} \tag{19}$$

Theorem 2 (Convergence) *The cost function $\mathcal{L}(W)$ in Equation (17) converges to a global minimum under the multiplicative update rule in Eq. (19).*

KKT requires $w_{i,j} \geq 0$ and $(\nabla \mathcal{L}(W)_{ij} w_{ij} = 0$. The first condition holds with non-negative initialisation of W. For the second condition, the gradient is: $\nabla \mathcal{L}(W) = -X^T X - \sum_j \mu_k W A_k + X^T X W + \sum_k \mu_k W D_k + aW + b + \gamma I$, and according to the second KKT condition, at convergence $W = W^*$ we have $(X^T X W^* + \sum_k \mu_k W^* D_k + aW^* + b + \gamma I)_{ij} W^*_{ij} - (X^T X + \sum_k \mu_k W^* A_k)_{ij} W^*_{ij} = 0$, which is identical to (19). That is, the multiplicative rule converges to a global optima.

Methods S3. On the interpretability of our model vs NMF models. Related to STAR Methods.

In this supplementary section, we would like to comment on the differences between our matrix decomposition model proposed for predicting the frequencies of drug side effects[7] and GSEM.

The interpretability of our matrix decomposition model and GSEM are different because the models are different. First, we would like to clarify our MF model[7] addresses a different question (predicting frequencies of drug side effects), uses additional data (frequency of side effects), and does not integrate side information about drugs or side effects.

The model formulation can explain the difference in model interpretability between the two models. In our matrix decomposition model, each drug and each side effect are represented as low dimensional feature vectors such that their dot product encodes the frequency of the drug side effect. Let u_i denote the learned feature vector of drug i, and v_j the feature vector of side effect j. Then, the prediction score between drug i and side effect j is:

$$\text{MF score}_{ij} = u_i \cdot v_j$$

We have shown that each element in the learned vectors of the model can be related to anatomical categories of drugs and disorder categories of side effects. The interpretability capabilities of this MF model are to examine, for a new drug, the activation weights on each component of the learned vectors in relationship with their established biological effects.

Instead, GSEM learns "drug similarities" (as captured in H) and "side effect similarities" (as captured in W). For the side effect self-representation model, the score between a drug i and a side effect j can be written as follows:

$$\text{score}_{ij} = \sum_{k \in \{A,B,C\}} W_{kj}$$

Where $\{A, B, C\}$ is the set of known side effects induced by drug i. This simple formulation of our model allows for interpretability: the predicted drug-side effect association can be explained by a learned similarity between the known side effects induced by drug i and the side effect j. Side effects which are more similar to side effect j will have a higher impact in the overall score. In Figure 7 of the main manuscript, we have illustrated an example of the learned weights for two side effects: (i) vision blurred; and (ii) myocardial infarction.

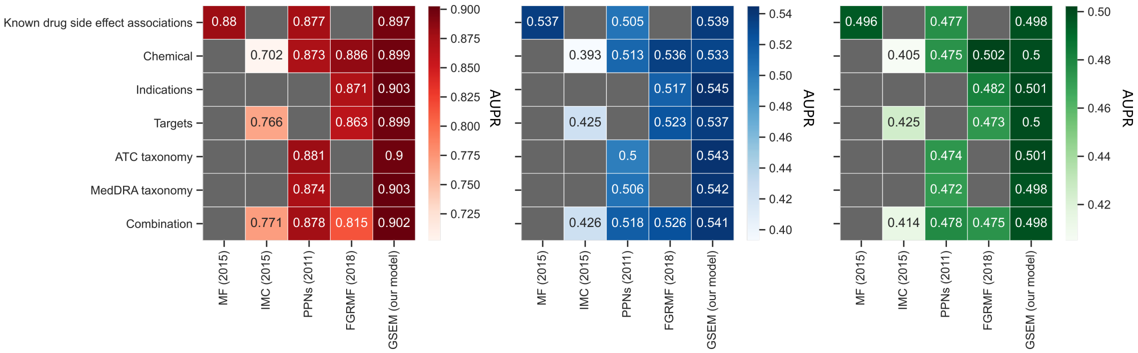

Fig. S1: Area Under the Precision Recall curve (AUPR) of each model type in each test set. Related to Figure 3. (red) Held-out test set containing clinical trials side effects; (blue) Postmarketing side effects from the SIDER database; (green) Postmarketing side effects from the OFFSIDES database.

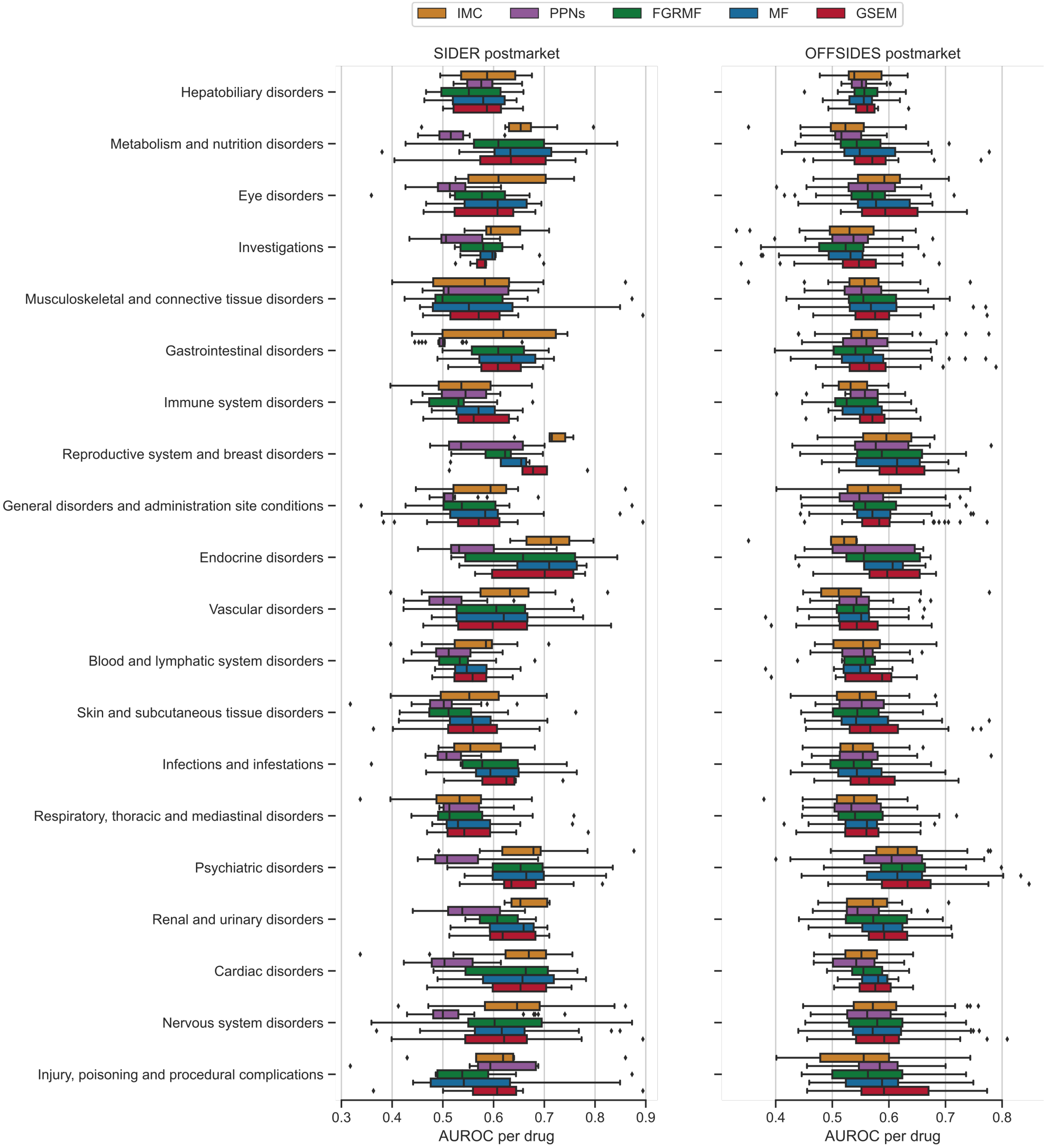

Fig. S2: Binary classification performance at predicting postmarket side effects in the SIDER and OFFSIDES databases. Related to Figure 3. AUROC computed for each drug by considering only the postmarketing side effects belonging to specific MedDRA category of disorders. (Left) SIDER postmarket set; (Right) OFFSIDES postmarket set.

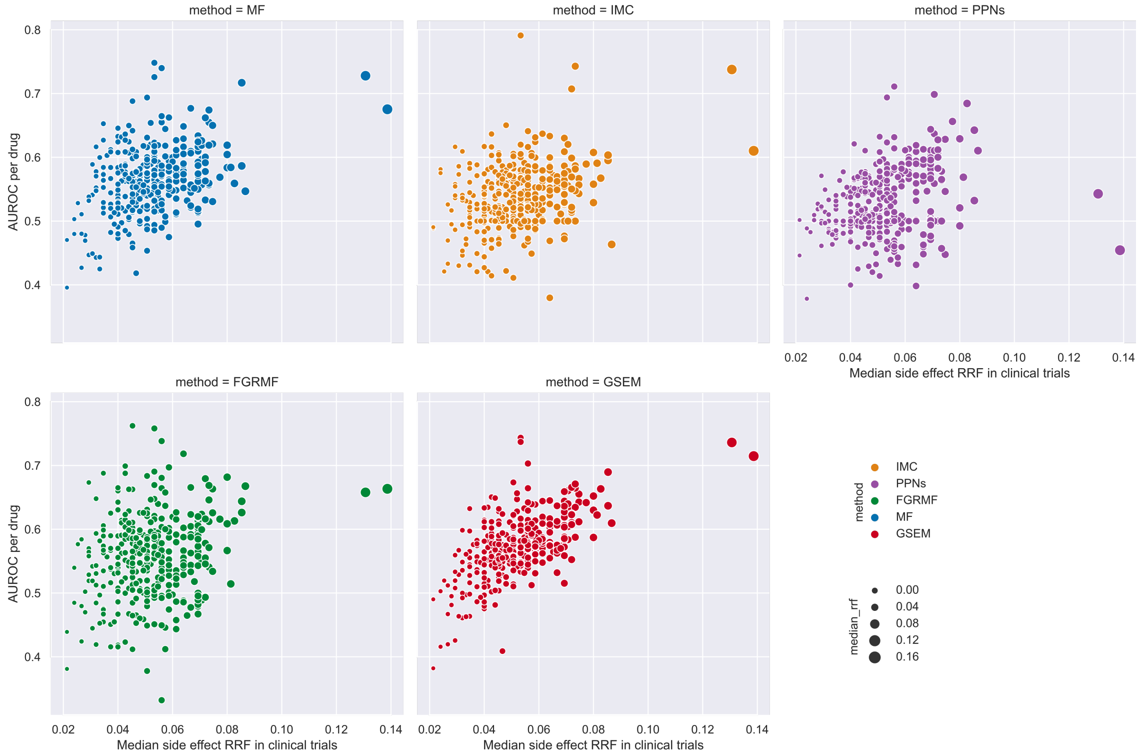

Fig. S3: Dependency between prediction performance and the median RRF values in the OFFSIDES postmarket test set. Related to Figure 5.

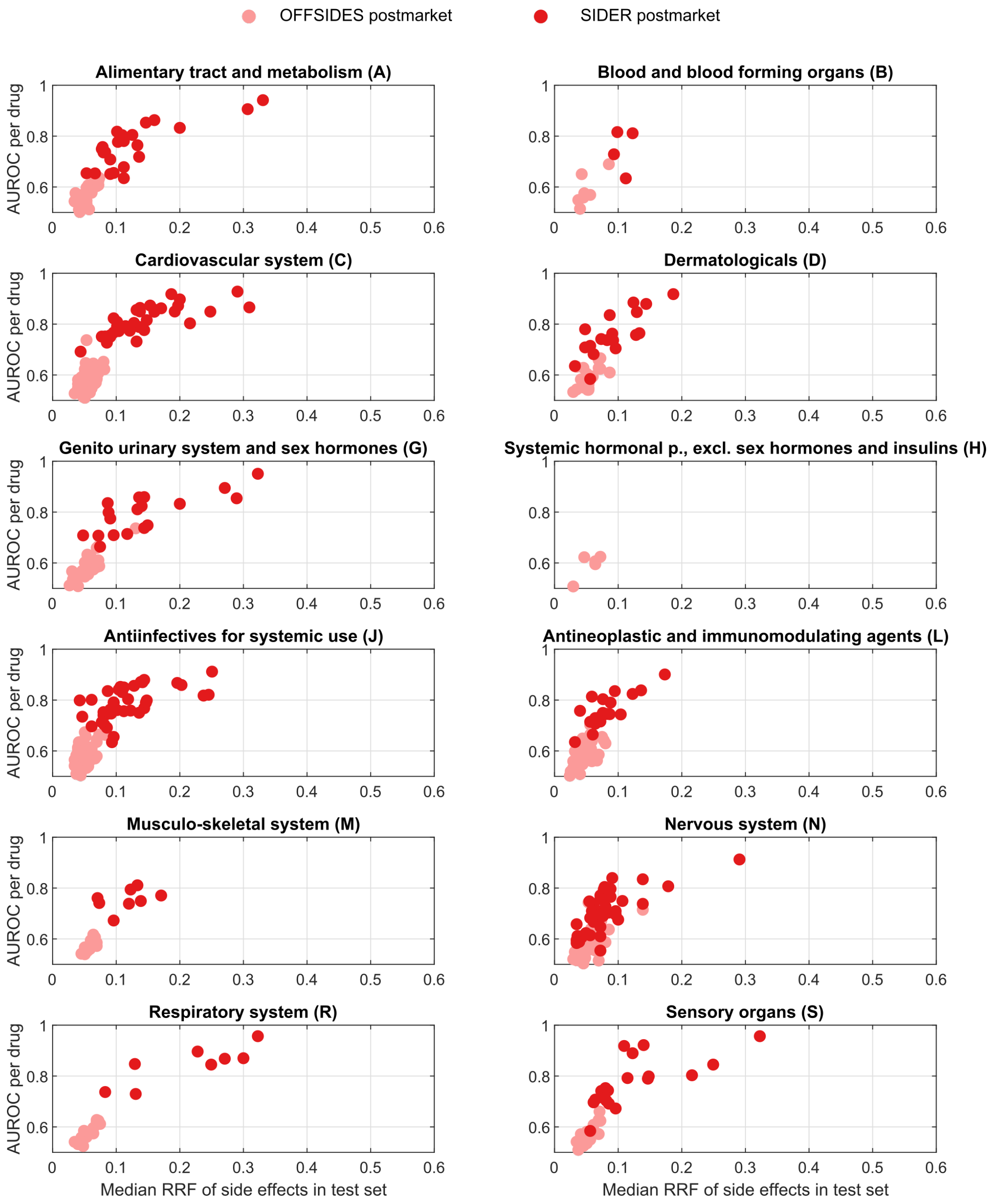

Fig. S4: Dependency between prediction performance for specific main drug Anatomical, Therapeutic and Chemical (ATC) categories and side effect RRF values in the postmarketing test sets. Related to Figure 5. Only drug categories with more than five drugs in the test sets are shown.

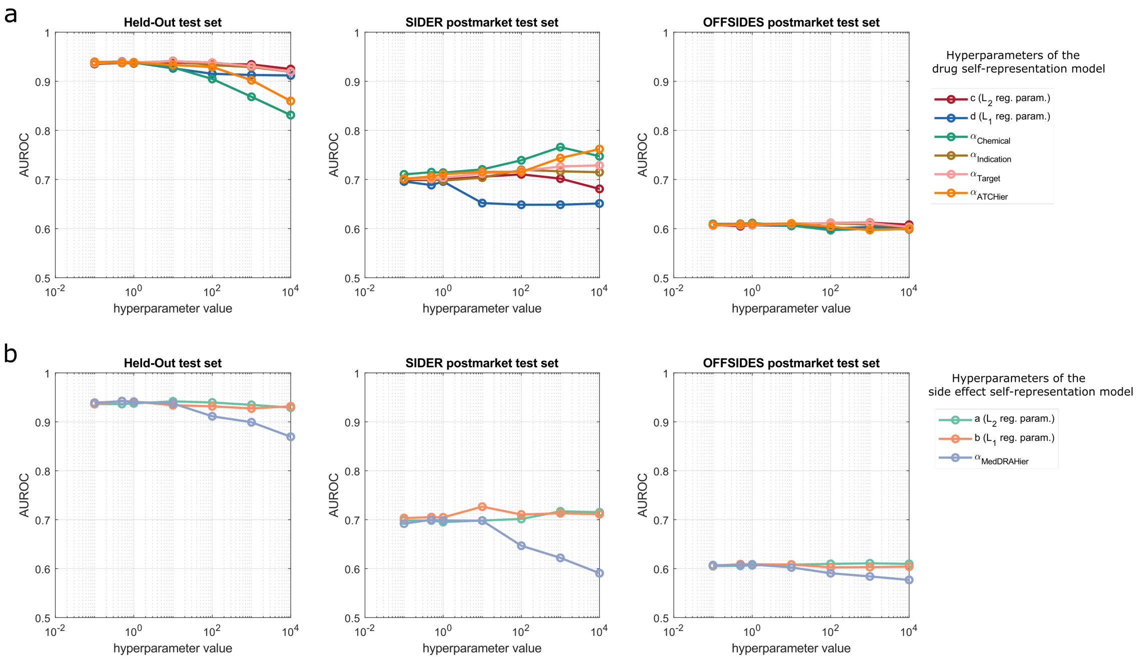

Fig. S5: Dependency between prediction performance and individual hyperparameters in GSEM. Related to Figure 3. (a) Drug self-representation model; (b) Side effect self-representation model. One hyperparameter at a time was change while all the others were set to zero.

References

Cami, A., Arnold, A., Manzi, S. and Reis, B. (2011), Predicting adverse drug events using pharmacological network models, Science translational medicine 3(114), 114ra127–114ra127.

Galeano, D. and Paccanaro, A. (2018), A recommender system approach for predicting drug side effects, in 2018 International Joint Conference on Neural Networks (IJCNN), IEEE, pp. 1–8.

Li, R., Dong, Y., Kuang, Q., Wu, Y., Li, Y., Zhu, M. and Li, M. (2015), Inductive matrix completion for predicting adverse drug reactions (adrs) integrating drug–target interactions, Chemometrics and Intelligent Laboratory Systems 144, 71–79.

Zhang, W., Liu, X., Chen, Y., Wu, W., Wang, W. and Li, X. (2018), Feature-derived graph regularized matrix factorization for predicting drug side effects, Neurocomputing 287, 154–162.

Berry, M. W., Browne, M., Langville, A. N., Pauca, V. P. and Plemmons, R. J. (2007), Algorithms and applications for approximate nonnegative matrix factorization, Computational statistics and data analysis 52(1), 155–173.

Boyd, S. and Vandenberghe, L. (2004), Convex optimization, Cambridge university press.

Galeano, D., Li, S., Gerstein, M. and Paccanaro, A. (2020), Predicting the frequencies of drug side effects, Nature communications 11(1), 1–14.
